# Supplementary material for: Modulation of antibiotic sensitivity and biofilm formation in Pseudomonas aeruginosa by interspecies signal analogues
Source: Nat Commun. 2019 May 27;10:2334. doi: 10.1038/s41467-019-10271-4 (PMC6536496; doi:10.1038/s41467-019-10271-4)
Supplement: Supplementary file 1 — Supplementary Information [file 41467_2019_10271_MOESM1_ESM.pdf]

Supplementary Information

Modulation of antibiotic sensitivity and biofilm formation in *Pseudomonas aeruginosa* by interspecies signal analogues

Shi-qi An, et al.

*Correspondence: s-q.an@soton.ac.uk (SA); m.valvano@qub.ac.uk (MAV); jltang@gxu.edu.cn (JLT)

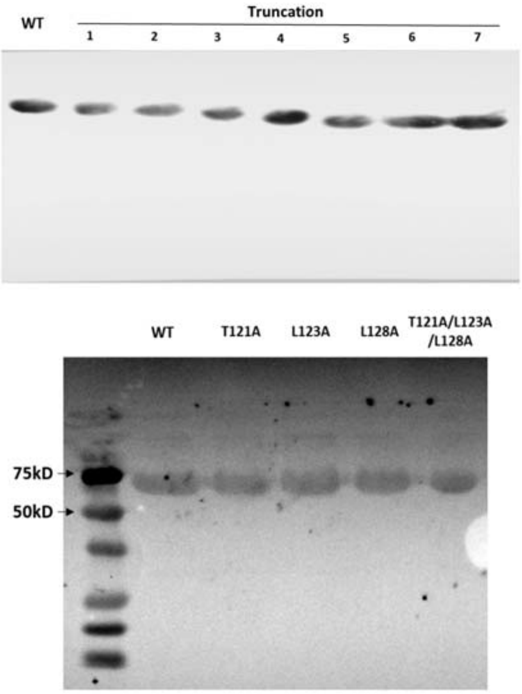

Supplementary Figure 1. Western blot analysis with an anti-His_6 antiserum (abcam, ab1187, 1:1000 dilution) shows that all variant and truncated PA1396 proteins are expressed in *Pseudomonas aeruginosa*. Top panel: Lanes: 1, PAO1 (PA1396His_6); 2, PAO1 (PA1396-035His_6); 3, PAO1 (PA1396-040His_6); 4, PAO1 (PA1396-082His_6); 5, PAO1 (PA1396-104His_6); 6, PAO1 (PA1396-114His_6); 7, PAO1 (PA1396-136His_6); 8, PAO1 (PA1396-143His_6). Bottom panel: Lanes: 1, PAO1 (PA1396His_6); 2, PAO1 (PA1396-T121A-His_6); 3, PAO1 (PA1396-L123A-His_6); 4, PAO1 (PA1396-L128A-His_6); 5, PAO1 (PA1396-T121A/L123A/L128A-His_6).

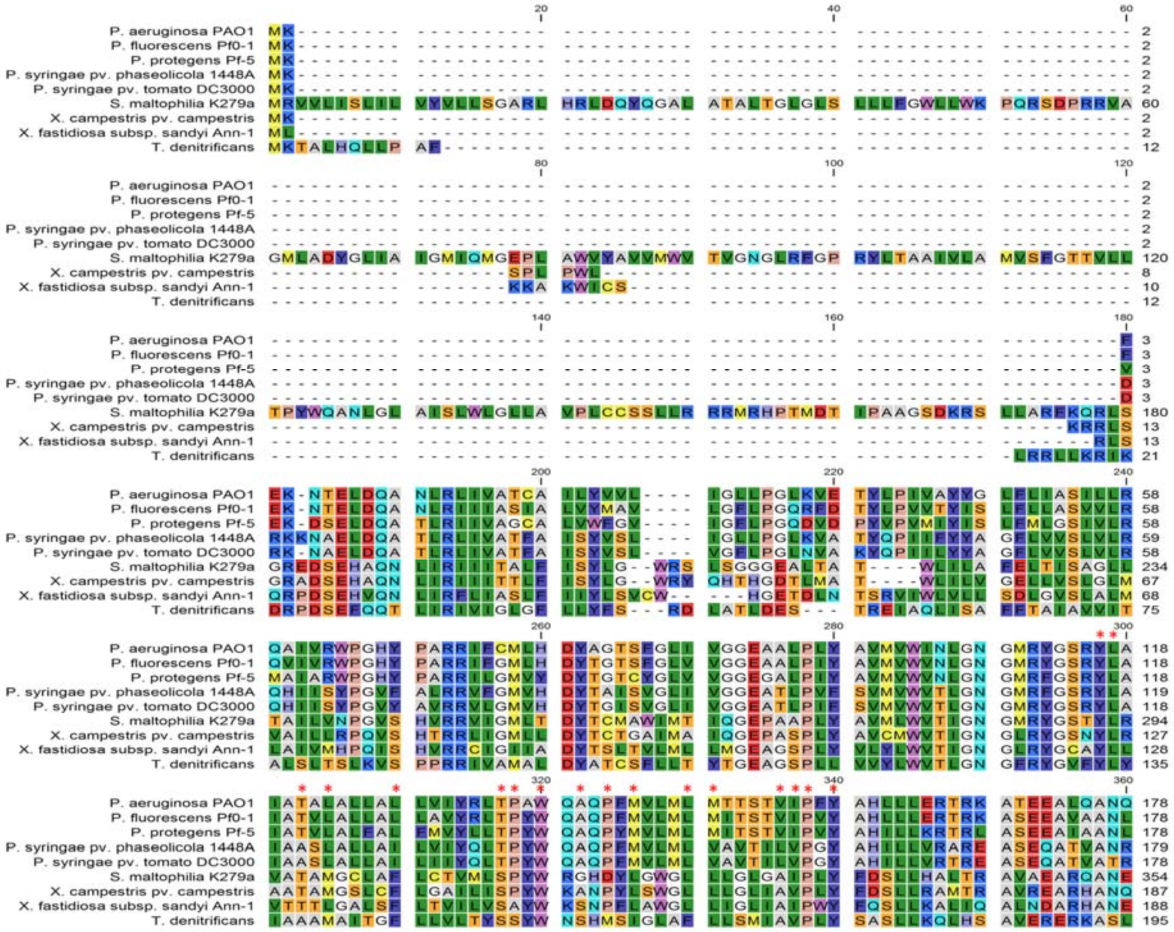

Supplementary Figure 2. Amino acid sequence comparisons between the input domain (amino acid residues 1–187) of RpfC from *Xcc* strain 8004, which is implicated in DSF perception, with input domains of sensor kinases from other bacteria including PA1396 of *P. aeruginosa*. The sequences were obtained from both complete and incomplete microbial genomes using the website at The Institute for Genomic Research (TIGR) at http://www.tigr.org, and were aligned using CLC workbench software. Residues with similar properties are boxed within the same colour. Residues with asterisks indicate those that were altered to alanine to test for a role in DSF perception.

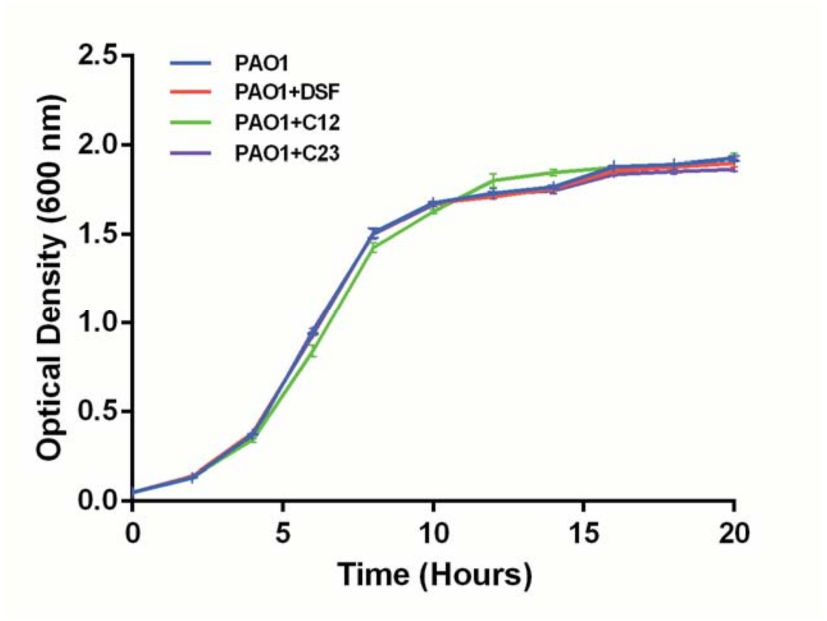

Supplementary Figure 3. Effects of DSF or DSF analogues on growth of *P. aeruginosa* PAO1 as measured by OD at 600 nm. Bacteria were grown in minimal medium at 37 °C with shaking in the presence of 10 μM of the different compounds. The observed values were not significantly different from the appropriate wild-type ($p<0.05$, ANOVA).

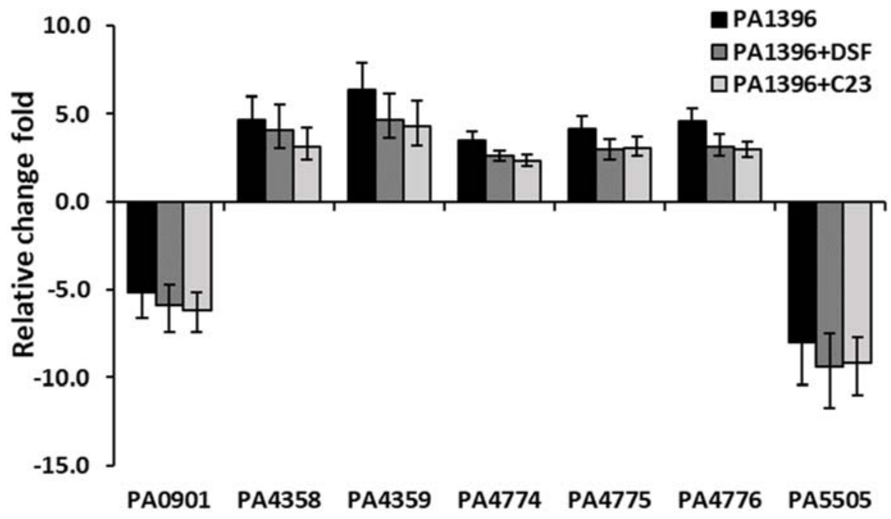

Supplementary Figure 4. Effects of addition of DSF or C23 on expression levels of selected genes in the *PA1396* mutant as measured by qRT-PCR. The genes studied (*PA0901*, *PA4358*, *PA4359*, *PA4774-PA4776* and *PA5505*) were previously implicated in the response of the wild-type to DSF. The qRT–PCR data were normalised to *proC* and are presented as the fold change with respect to the wild-type for each gene. Data (means ± standard deviation) are representative of three independent experiments. The observed values were not significantly different from the appropriate wild-type ($p<0.05$, Student's t test).

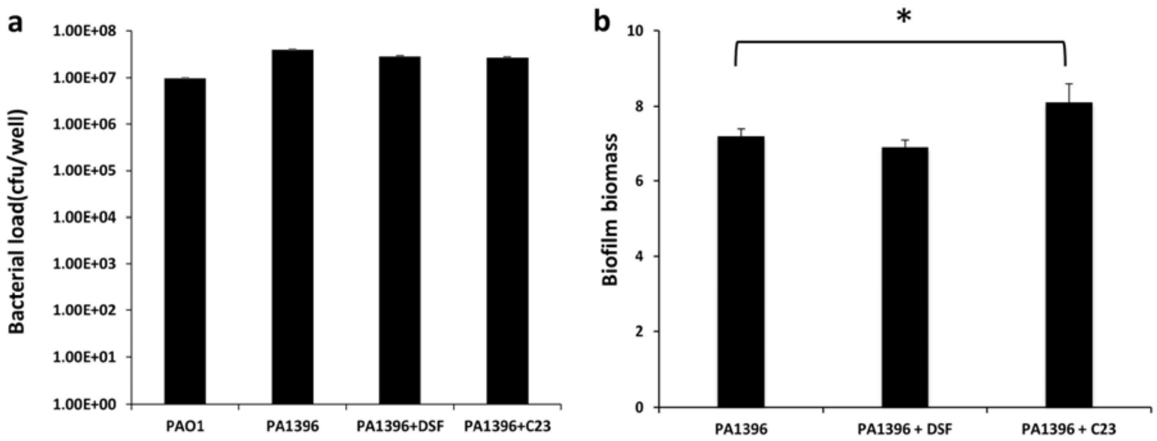

Supplementary Figure 5. (a) Effect of DSF and C23 on attachment of the *PA1396* mutant to CFBE epithelial cells. For these experiments, compounds (0.5 μM) were added to the co-culture at 1h and bacterial attachment to the CFBE epithelial cells was measured after 24 h (see Materials and Methods). (b) Effect of DSF and C23 (0.5 μM) on attachment of the *PA1396* mutant on to a glass surface as assessed by crystal violet staining. Biofilm biomass is measured as a ratio of absorbance at 550 and 600 nm. Data (means ± standard deviation) are representative of three independent experiments. The means and standard deviations of triplicate measurements are shown. A p value of <0.05 was considered statistically significant and is designated in the figures with an asterisk. Double asterisks indicate p values of <0.01.

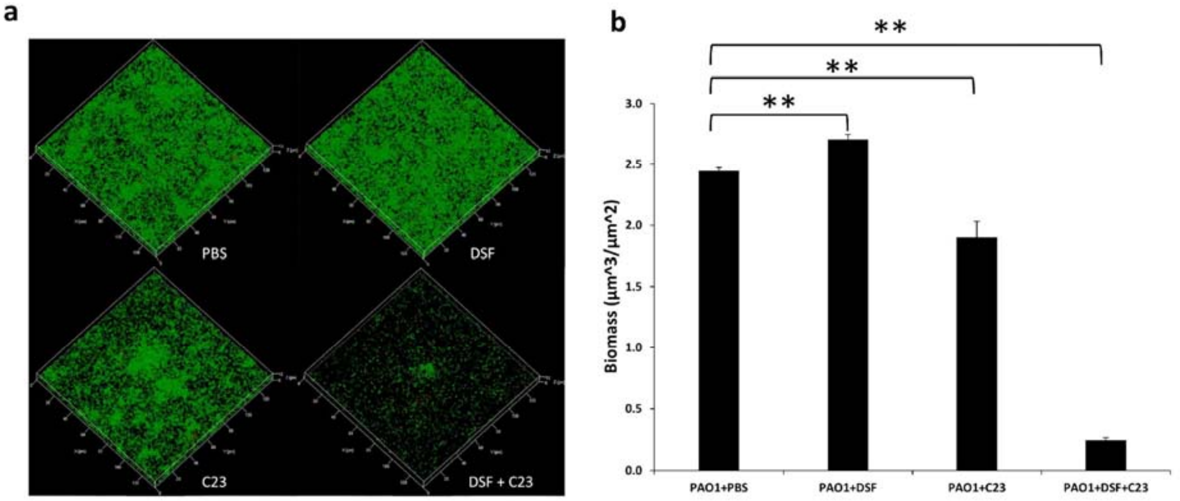

Supplementary Figure 6. Effect of DSF and C23 on biofilm formation of PAO1 developed in µ-well chambers. For these experiments, compounds (0.5 µM) were added to in ABTGC media and biofilms were developed for 16 h in µ-well chambers. (a) Confocal laser scanning microscopy images of biofilms. (b) The biofilm biomass was quantified using COMSTAT. Data are presented as the average of four technical replicates, with error bars representing the standard deviation of the data. A p value of <0.05 was considered statistically significant and is designated in the figures with an asterisk. Double asterisks indicate p values of <0.01.

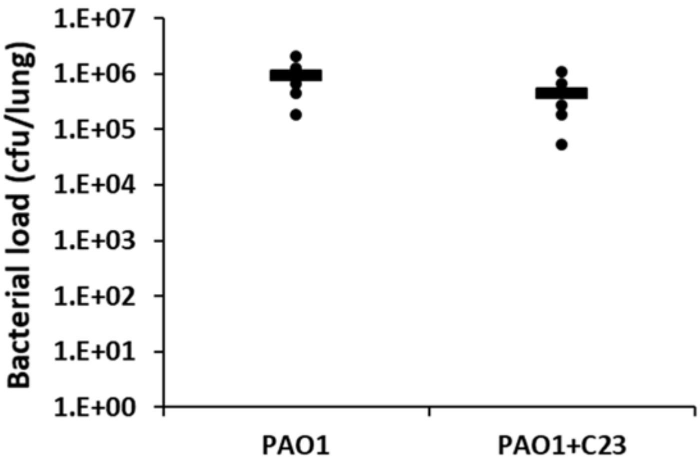

Supplementary Figure 7. Administration of C23 to mouse airway infection by *P. aeruginosa* PAO1. Here C57BL/6 mice were infected intranasally with 1×10^7 CFU PAO1 and treated by inhaling PBS with or without 50 μM of C23. After 24 hours infection, the mice were harvested, and bacterial loads were determined in lung homogenates. Each data point shows the results from an individual mouse. The observed values were not significantly different from the appropriate wild-type ($p<0.05$, Student's t test).

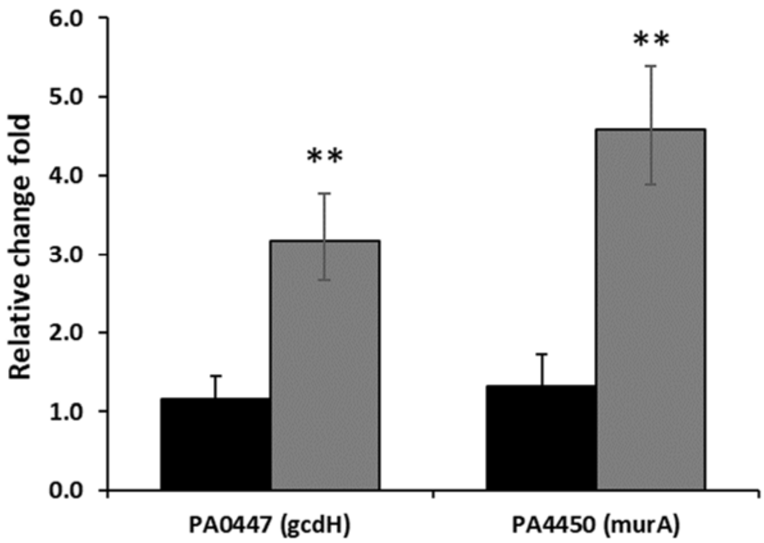

Supplementary Figure 8. Effect of addition of C23 to wild-type *P. aeruginosa* on expression levels of selected genes implicated in virulence and biofilm formation as measured by qRT-PCR. Transcript levels of *gcdH* (PA0447) and *murA* (PA4450) were examined. The qRT–PCR data were normalised to *proC* and is presented as the fold change with respect to the wild-type for each gene. Data (means ± standard deviation) are representative of three independent experiments. A p value of <0.05 was considered statistically significant and is designated in the figures with an asterisk. Double asterisks indicate p values of <0.01.

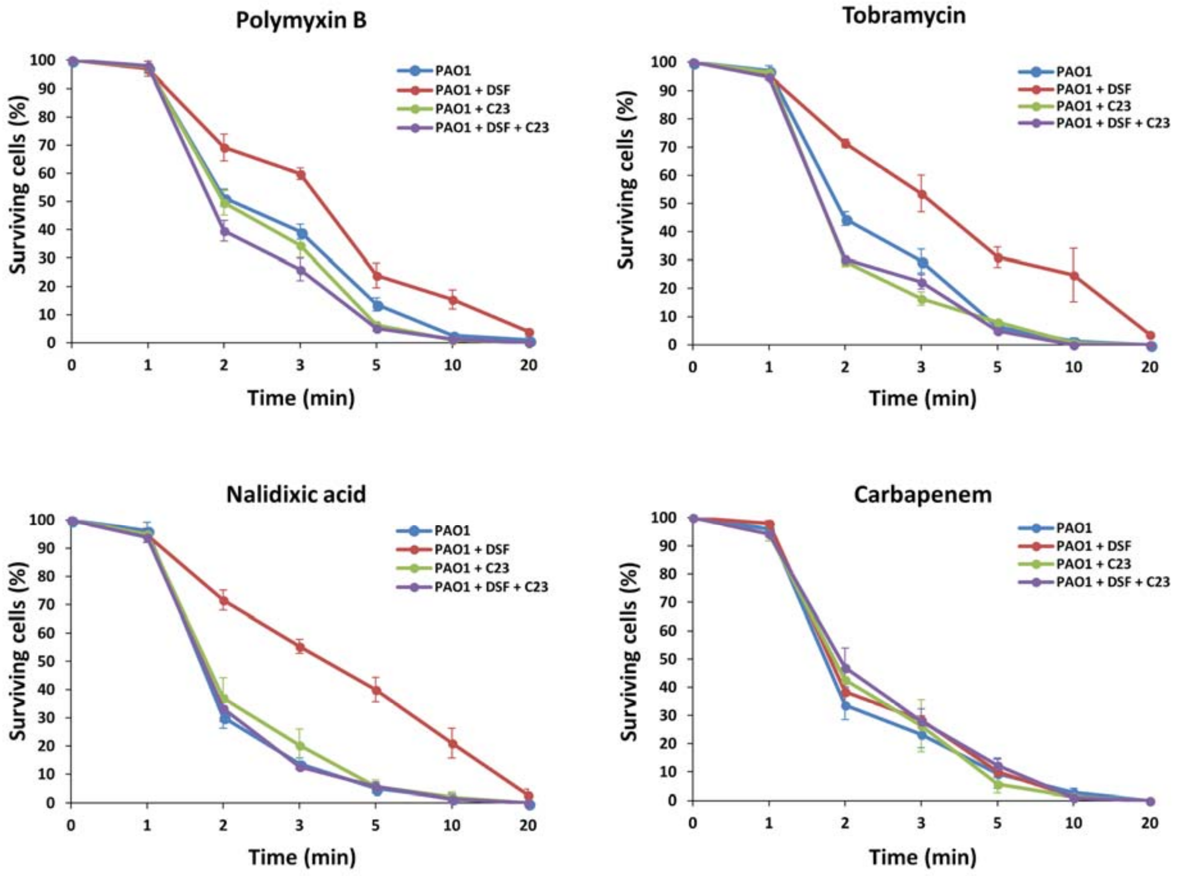

Supplementary Figure 9. Effect of addition of DSF and C23 alone or in combination on tolerance of *P. aeruginosa* PAO1 to antibiotics. Time-courses of killing of *P. aeruginosa* by polymyxin B (4 μg ml^{-1}), tobramycin (2 μg ml^{-1}), nalidixic acid (10 μg ml^{-1}) and carbapenem (10 μg ml^{-1}) were established for bacteria suspended in sodium phosphate buffer. Bacteria for these experiments were grown in BM2 medium with glucose supplemented with 2 mM Mg^{2+}. DSF and C23 were added to these cultures to a final concentration of 50 μM. Data (means ± standard deviation) are representative of three independent experiments. The observed values of addition of DSF and C23 were significantly different from the appropriate wild-type with addition of DSF in polymyxin B, Tobramycin, Nalidixic acid treatment. ($p<0.05$, ANOVA).

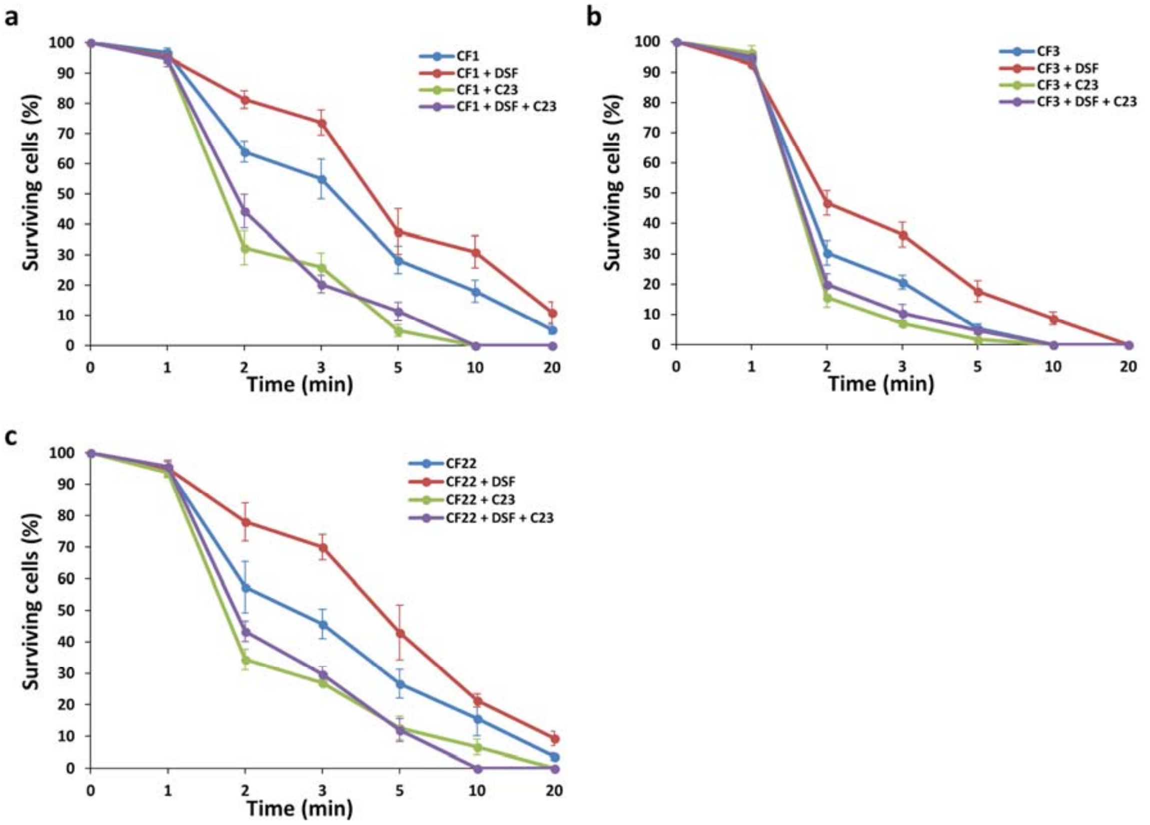

Supplementary Figure 10. Effect of addition of DSF analogues on the tolerance to polymyxin B of selected *P. aeruginosa* clinical isolates CF1 (a), CF3 (b) and CF22 (c). Time-courses of killing by 4 µg ml^{-1} polymyxin B were established for bacteria suspended in sodium phosphate buffer. Bacteria for these experiments were grown in BM2 medium with glucose supplemented with 2 mM Mg^{2+}. When required, DSF was added to these cultures to a final concentration of 50 µM. Data (means ± standard deviation) are representative of three independent experiments. The observed values of addition of DSF and C23 were significantly different from the appropriate wild-type addition of DSF in all cases ($p<0.05$, ANOVA).

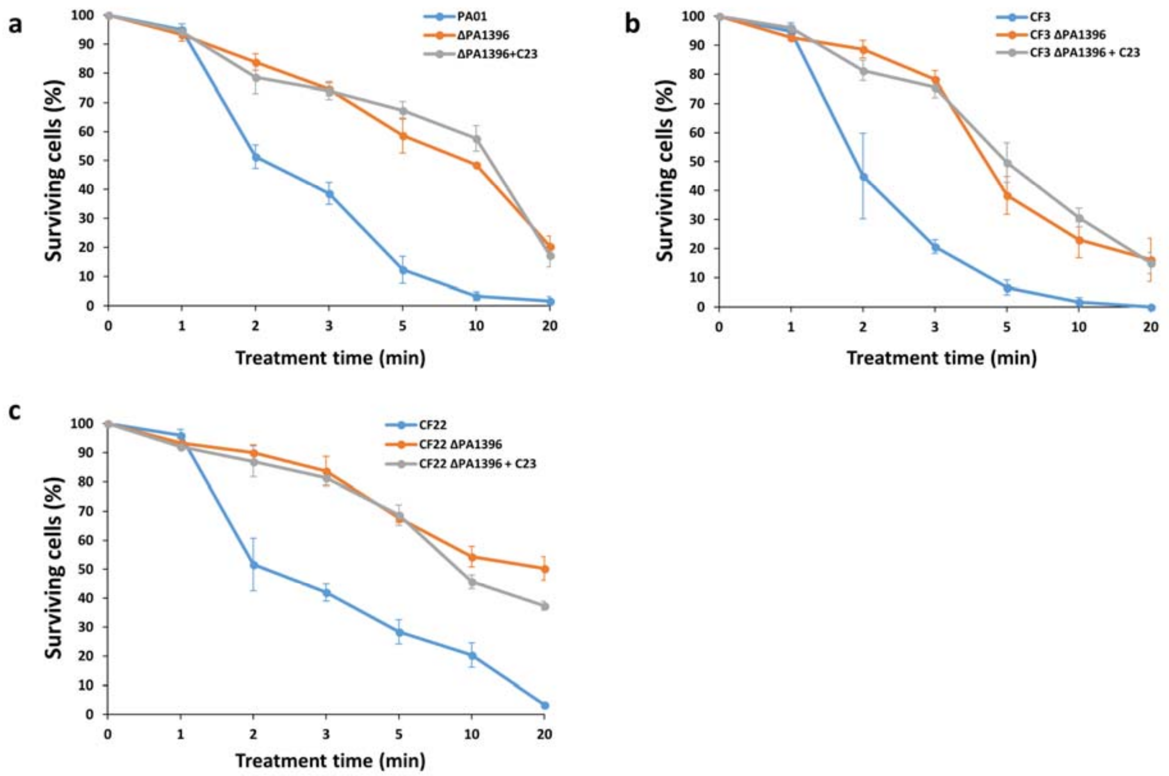

Supplementary Figure 11. Effects of administration of C23 to clinical isolates of *P. aeruginosa* carrying a *PA1396* mutated gene on tolerance to polymyxins. Time-courses of killing of *P. aeruginosa* PAO1-*PA1396* (a), CF3-*PA1396* (b), CF22-*PA1396* (c) by 2 μg ml^{-1} polymyxin B were established for bacteria suspended in sodium phosphate buffer. Data (means ± standard deviation) are representative of three independent experiments. The observed values of ΔPA1396 and C23 were not significantly different from the ΔPA1396 in all cases ($p>0.05$, ANOVA).

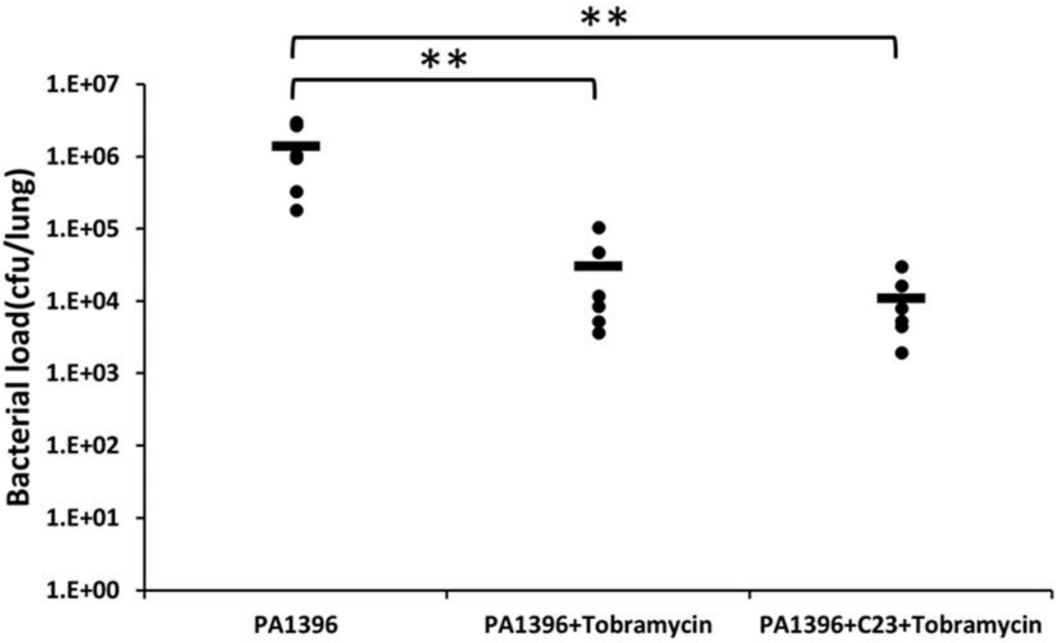

Supplementary Figure 12. Effects of administration of C23 on infection of the *PA1396* mutant strain of *P. aeruginosa* from the mouse airway in the presence of tobramycin**.** C57BL/6 mice were infected intranasally with 1 × 10^7 CFU *PA1396* mutant and treated by inhaling PBS with or without 50 μM C23. After 24 hours infection, the mice were harvested, and bacterial loads were determined in lung homogenates. Each data point shows the results from an individual mouse. A *p* value of <0.05 was considered statistically significant and is designated in the figures with an asterisk. Double asterisks indicate *p* values of <0.01.

Supplementary Table 1. Enzyme activities of PA1396-LacZ and PA1396-PhoA constructs.

**Fusion**[a]	**LacZ activity**[b]	**PhoA activity**[c]
E4	N.D.[d]	62 ± 5.6
N6	N.D.	49 ± 1.9
G66	N.D.	39 ± 2.2
A70	N.D.	57 ± 3.3
A137	N.D.	28 ± 12.1
Q139	N.D.	67 ± 4.9
Q141	N.D.	22 ± 9.1
V37	490 ± 50	N.D.
E38	730 ± 30	N.D.
G107	910 ± 70	N.D.
G109	870 ± 120	N.D.
L205	580 ± 40	N.D.
T240	640 ± 60	N.D.
L300	720 ± 80	N.D.
L381	850 ± 120	N.D.

[a] The β-galactosidase and alkaline phosphatase activities were measured in strain E. coli containing the plasmid encoded PA1396-lacZ or PA1396-phoA fusion. All enzyme assays were done in triplicate.

[b] LacZ activity is expressed as micromoles of ONPG hydrolyzed per minute per milligram of protein. The LacZ activity of strain without fusions is equal to zero.

[c] PhoA activity is expressed as micromoles of pNPP hydrolyzed per minute per microgram of protein. The PhoA activity of strain without fusions is equal to zero.

[d] N.D. – Not detected.

Supplementary Table 2. Auto-phosphorylation of PA1396 in response to DSF and analogues.

Protein	**- DSF**	**+ DSF**	**+ C23**	**+ DSF + C23**
**PA1396**	1 (± 0.02)	3.5 (± 0.13)	1 (± 0.67)	1.3 (± 0.15)
**PA1396-T121A/L123A/L128A**	1 (± 0.07)	1 (± 0.21)	1 (± 0.31)	1 (± 0.09)

Densitometric quantification of level of phosphorylation in protein bands was done using the Image J software. The levels of protein phosphorylation were quantified as mean ± s.d. (n = 6) and are presented as values relative to the value seen with PA1396 alone (which was set at 1).

Supplementary Table 3. *P. aeruginosa* genes differentially regulated during infection in the presence of DSF or DSF with C23.

ORF[a]	Category or class or gene/protein name[a]	Fold changes[b]	
		WT+DSF	WT+DSF+C23
PA0162	histidine porin OpdC	-1.25	
PA0281	sulfate transporter CysW	1.44	1.35
PA0283	sulfate-binding protein	1.45	1.38
PA0284	hypothetical protein	1.48	1.41
PA0494	acetyl-CoA carboxylase biotin carboxylase subunit		-1.25
PA0495	hypothetical protein		-1.27
PA0512	hypothetical protein		-1.29
PA0513	transcriptional regulator		-1.30
PA0524	nitric-oxide reductase subunit B	-1.27	
PA0525	dinitrification protein NorD		-1.25
PA0534	hypothetical protein		1.26
PA0612	repressor PtrB	1.36	
PA0613	hypothetical protein	1.41	
PA0614	hypothetical protein	1.41	
PA0615	hypothetical protein	1.34	
PA0616	hypothetical protein	1.29	
PA0617	bacteriophage protein	1.38	
PA0618	bacteriophage protein	1.32	
PA0619	bacteriophage protein	1.27	
PA0620	bacteriophage protein	1.34	
PA0621	hypothetical protein	1.34	
PA0622	bacteriophage protein	1.30	
PA0624	hypothetical protein	1.25	
PA0625	hypothetical protein	1.27	
PA0626	hypothetical protein	1.26	
PA0627	hypothetical protein	1.36	
PA0628	hypothetical protein	1.28	
PA0629	hypothetical protein	1.28	
PA0630	hypothetical protein	1.35	
PA0631	hypothetical protein	1.31	
PA0632	hypothetical protein	1.31	
PA0633	hypothetical protein	1.28	
PA0634	hypothetical protein	1.36	
PA0635	hypothetical protein	1.30	
PA0636	hypothetical protein	1.30	
PA0637	hypothetical protein	1.37	
PA0638	bacteriophage protein	1.29	
PA0639	hypothetical protein	1.32	
PA0641	bacteriophage protein	1.27	

PA0643	hypothetical protein	1.27	
PA0644	hypothetical protein	1.48	
PA0645	hypothetical protein	1.26	
PA0802	hypothetical protein		1.27
PA0806	hypothetical protein	1.27	
PA0887	acetyl-CoA synthetase	1.28	
PA0910	hypothetical protein	1.42	
PA0911	hypothetical protein	1.39	
PA1183	C4-dicarboxylate transporter DctA		1.25
PA1318	cytochrome o ubiquinol oxidase subunit I		-1.32
PA1319	cytochrome o ubiquinol oxidase subunit III		-1.29
PA1320	cytochrome o ubiquinol oxidase subunit IV		-1.26
PA1325	hypothetical protein		1.42
PA1326	threonine dehydratase	1.31	1.53
PA1425	ABC transporter ATP-binding protein		1.26
PA1600	cytochrome C		-1.32
PA1601	aldehyde dehydrogenase		-1.34
PA1602	oxidoreductase		-1.32
PA1709	translocator outer membrane protein PopD	-1.28	
PA1797	hypothetical protein	1.68	1.63
PA2009	homogentisate 1,2-dioxygenase		1.35
PA2018	multidrug efflux protein	1.36	1.42
PA2019	periplasmic multidrug efflux lipoprotein	1.34	1.39
PA2204	ABC transporter	1.43	1.28
PA2322	gluconate permease		-1.30
PA2357	NADH-dependent FMN reductase MsuE	1.39	1.43
PA2358	hypothetical protein	1.51	1.50
PA2485	hypothetical protein		1.31
PA2655	hypothetical protein	1.68	1.71
PA2659	hypothetical protein	1.26	
PA2663	psl and pyoverdine operon regulator, PpyR	-1.29	-1.28
PA2664	nitric oxide dioxygenase	-1.33	-1.34
PA3190	sugar ABC transporter substrate-binding protein		-1.28
PA3445	hypothetical protein	1.28	
PA3446	NAD(P)H-dependent FMN reductase	1.57	1.46
PA3450	antioxidant protein	1.49	1.38
PA3530	hypothetical protein		1.48
PA3780	hypothetical protein		1.31
PA3841	exoenzyme S	-1.28	
PA3875	hypothetical protein	-1.28	
PA3876	nitrite extrusion protein 2	-1.39	
PA3931	hypothetical protein	1.49	1.39
PA3932	transcriptional regulator	1.39	
PA4138	tyrosyl-tRNA synthetase		1.28
PA4193	ABC transporter permease	1.30	

PA4194	ABC transporter permease	1.27	
PA4195	ABC transporter	1.50	1.31
PA4290	chemotaxis transducer	1.31	1.25
PA4359	ferrous iron transporter A	1.29	1.27
PA4599	resistance-nodulation-cell division (RND) multidrug efflux membrane fusion protein MexC		1.31
PA4685	hypothetical protein		-1.55
PA4777	two-component regulator system signal sensor kinase PmrB	1.34	
PA4823	hypothetical protein	1.25	1.43
PA4824	hypothetical protein		1.26
PA4825	Mg(2+) transport ATPase	1.51	1.66
PA5445	coenzyme A transferase		1.26
PA5470	peptide chain release factor-like protein		1.46
PA5471	hypothetical protein		1.33

[a] From *P. aeruginosa* genome website, http://www.pseudomonas.com.

[b] Regulation (*n*-fold) of genes differentially expressed during *P. aeruginosa* infection with DSF or DSF + C23 compared to wild-type; a positive number indicates an up-regulation of the gene and a negative number indicates a down-regulation of the gene.

Supplementary Table 4. Bacterial strains and plasmids used in this study.

Strain	Relevant genotype or description	Reference or source
Pseudomonas aeruginosa		
PAO1	Wild-type	www.pseudomonas.com
PA1396	PA1396::Gm^r mutant of PAO1 derivative created using pEX18Gm	1
PA1396	PA1396 delection mutant of PAO1 derivative created using pEX18Gm	2
PAO1 *pmr-gfp* fusion	GFP expression from the reporter fusion	3
PA1396 *pmr-gfp* fusion	GFP expression from the reporter fusion	2
CF1	CF patient from University College Cork	2
CF3	CF patient from University College Cork	2
CF22	CF patient from University College Cork	2
CF1-PA1396	PA1396::Gm^r mutant of CF3 derivative created using pEX18Gm	2
CF3-PA1396	PA1396::Gm^r mutant of CF3 derivative created using pEX18Gm	2
CF22-PA1396	PA1396::Gm^r mutant of CF22 derivative created using pEX18Gm	2
Xanthomonas campestris		
8004	Wild type; Rif^r	4
8523	*rpfF* mutant; DSF–	4
Escherichia coli		
JM109	endA recA1 gyrA96 thi-1 hsdR17 lacIqZM15 relA1	Promega
BL21 (DE3)	*fhuA2 [lon] ompT gal (λ DE3) [dcm] ΔhsdS* *λ DE3 = λ sBamHIo ΔEcoRI-B int::(lacI::PlacUV5::T7 gene1) i21 Δnin5*	Sigma
DH5α	*end*A, *hsd*R, *sup*E44, *thi-1, rec*A1, *gyr*A, *rel*AΔ, (*lacZYA-argF*), U169 [Φ80 dlacΔ(*lacZ*) M15] *phoA*	Promega
**Plasmids**		
pEX18Gm	Broad-host-range allelic exchange vector; Gm^r	5
pBBR1MCS	Broad host range cloning vector, Cm^r	5

pBAD/Myc-HisA	C-terminal polyhistidine (6xHis) tag and *c-myc* epitope expression vector	Life technologies
PA1396FL-pBAD/Myc-His	pBAD-MycHis expressing PA1396	This study
PA1396T-pBAD/Myc-His	pBAD-MycHis expressing truncation (1-250aa) of PA1396	This study
pRMCD28	*E. coli pho*A low-copy number topology vector. Amp^R	4
pRMCD70	*E. coli lac*Z low-copy number topology vector. Amp^R	4
pET47b+	Expression vector. Amp^R	Novagen

Supplementary Table 5. Primers and synthesized fragments of DNA used in this study.

Primer	Comment	Sequence (5' to 3')
P1396F<br>P1396R	Primers used to construct PA1396 construct	5'-CTCGAGATGAAGTTCGAGAAGAATACC-3'<br>5'-AAGCTTGGAAGCGTGGCTGGCGGT-3'
P1-35F<br>P1-35R	Primers used to construct PA1396 1-35 truncation	5'-CTCGAGATGGCCTGCTGCCCGGCCTG-3'<br>5'-AAGCTTGGAAGCGTGGCTGGCGGT-3'
P1-40F<br>P1-40R	Primers used to construct PA1396 1-40 truncation	5'-CTCGAGATGCTGCCGATCGTTGCCTACTA-3'<br>5'-AAGCTTGGAAGCGTGGCTGGCGGT-3'
P1-82F<br>P1-82R	Primers used to construct PA1396 1-82 truncation	5'-CTCGAGATGACCTCGTTCGGCCTGATC-3'<br>5'-AAGCTTGGAAGCGTGGCTGGCGGT-3'
P1-104F<br>P1-104R	Primers used to construct PA1396 1-104 truncation	5'-CTCGAGATGAACCTGGGCAACGGCATGCGCTA-3'<br>5'-AAGCTTGGAAGCGTGGCTGGCGGT-3'
P1-114F<br>P1-114R	Primers used to construct PA1396 1-114 truncation	5'-CTCGAGATGCGCTACCTGGCCATCGCCACCG-3'<br>5'-AAGCTTGGAAGCGTGGCTGGCGGT-3'
P1-136F<br>P1-136R	Primers used to construct PA1396 1-336 truncation	5'-CTCGAGATGGCCTGGCAGGCTCAGCCGTTCAT-3'<br>5'-AAGCTTGGAAGCGTGGCTGGCGGT-3'
P1-143F<br>P1-143R	Primers used to construct PA1396 1-143 truncation	5'-CTCGAGATGGTGCTGATGCTGATGACCACC-3'<br>5'-AAGCTTGGAAGCGTGGCTGGCGGT-3'
**Construct**	**Comment**	**DNA fragment synthesized**
pPA1396	PA1396 cloned into pBAD/*Myc*-His	CTCGAGGAAGTTCGAGAAGAATACCGAGCTGGACCAG<br>GCCAACCTGCGACTGATCGTGGCCACCTGCGCGATCC<br>TCTACGTGGTGCTGATCGGCCTGCTGCCCGGCCTGAAG<br>GTCGAGACCTACCTGCCGATCGTTGCCTACTACGGCCT<br>GTTCCTGATCGCCTCCATACTGCTGCGCCAGGCCATCG<br>TGCGCTGGCCGGGGCACTACCCGGCGCGGCGGATCTT<br>CTGCATGCTGCACGACTACGCCGGCACCTCGTTCGGCC<br>TGATCGTGGGCGGCGAGGCAGCGCTGCCGCTGTACGC<br>GGTGATGGTCTGGATCAACCTGGGCAACGGCATGCGC<br>TACGGCTCGCGCTACCTGGCCATCGCCACCGCCCTGGC<br>GCTGCTCGCGCTACTGGTCATCTATCGACTGACCCCGG<br>CCTGGCAGGCTCAGCCGTTCATGGTGCTGATGCTGATG<br>ACCACCAGTACCGTCATTCCCTTCTACGCGCACCTCCT<br>GCTGGAGCGCACGCGCAAGGCCACCGAGGAAGCGTTG<br>CAGGCGAACCAGGAGAAATCGCGCCTGCTGGCCCAGG<br>CCAGCCACGACCTGCGCCAGCCGATCCACTCCATCGG<br>CCTGTTCACCGCCTGCCTGCGCGACGCCCGCCTGGGCG<br>ACGAGGAACGGCGCCTGGTGGACAACATCGACCGCTC<br>GCTGCTCAACGTCTCGCAACTGTTCCGCTCCATTCTCG<br>ATCTCTACACCCTCGACAACGGCCGGCTCCAACCCAA<br>GCAGGAGAACGTCCACCTGGGCGAGTTGCTGCGCGAC<br>CTGGTCCGGCGCAACGCCGAAGCGGCGCGCTGGGCCG<br>GGGTGGAACTGCGCCTGCGCCCTTGCCGCCTGTGGAC<br>GCGAACCGATCCGGGGCTGCTGTCGACCATGCTGCAG<br>AACCTGCTCTCCAACAGCTTCAAGTACGCCGCCGAGC<br>GCCCGCTGCTGATCGGCGTGCGGCGGCGAGGCGACGG<br>CCTGGCCGTAGCCATCTACGACCAGGGCCGGGGGATC<br>GCGGAGGAACACCTGCCGCGGGTGTTCGAGGAGTTCT<br>ACCGGGTACGCGAGACGCGCGACCGCGACGTCGAGG<br>GAATCGGCCTGGGGCTGTCCATCGTCCGCCGCCTGGG<br>GCAGTTGACCGGGATCGAGGTGACGCTGCGCTCGCGG<br>GTCGGACGCGGCACCGCGGTGACCCTGCACGGCCTGC<br>CGGCGGTCGCCGCGCAAGCCCTGCCCCGCCGCGACGA<br>TCCCCTGCAGGCCGGCCTGCTCACCGGCTTGCGGGTGT<br>GCCTGGTGGAAGATGATCGCAACGTCCTGCGCGCCAC<br>CTCGGCGTTGCTCGAACGCTGGGGCTGCACGGTCCAG<br>GCGGAAACCGAGGCGGACGGCTGGCGAACCGATTGC<br>GACATCCTCGTCGTCGACTACGACCTCGGCCCCCACGC<br>CTCCGGCGTCGAGTGCATCGAGCGGGTACGGCGGCAA<br>CGCGGAGAGGCGATACCGGCGCTGGTGATCAGCGGCC<br>ACGACATCGAGCGTATCCAGGCCAGCGTCGAAGACAC<br>CGACATCGCCCTGCTCTCCAAGCCCGTGCGCCCCACG<br>GAATTGCGCGCCACCCTGCGCGCCCTGCGCGAGCGCC

		CGGTGACCGCCAGCCACGCTTCCAAGCTT
pPA1396trun	PA1396 truncation cloned into pBAD/*Myc*-His	CTCGAGGAAGTTCGAGAAGAATACCGAGCTGGACCAG GCCAACCTGCGACTGATCGTGGCCACCTGCGCGATCC TCTACGTGGTGCTGATCGGCCTGCTGCCCGGCCTGAAG GTCGAGACCTACCTGCCGATCGTTGCCTACTACGGCCT GTTCCTGATCGCCTCCATACTGCTGCGCCAGGCCATCG TGCGCTGGCCGGGGCACTACCCGGCGCGGCGGATCTT CTGCATGCTGCACGACTACGCCGGCACCTCGTTCGGCC TGATCGTGGGCGGCGAGGCAGCGCTGCCGCTGTACGC GGTGATGGTCTGGATCAACCTGGGCAACGGCATGCGC TACGGCTCGCGCTACCTGGCCATCGCCACCGCCCTGGC GCTGCTCGCGCTACTGGTCATCTATCGACTGACCCCGG CCTGGCAGGCTCAGCCGTTCATGGTGCTGATGCTGATG ACCACCAGTACCGTCATTCCCTTCTACGCGCACCTCCT GCTGGAGCGCACGCGCAAGGCCACCGAGGAAGCGTTG CAGGCGAACCAGGAGAAATCGCGCCTGCTGGCCCAGG CCAGCCACGACCTGCGCCAGCCGATCCACTCCATCGG CCTGTTCACCGCCTGCCTGCGCGACGCCCGCCTGGGCG ACGAGGAACGGCGCCTGGTGGACAACATCGACCGCTC GCTGCTCAACGTCTCGCAACTGTTCCGCTCCATTCTCG ATCTCTACACCCTCGACAACGGCCGGCTCCAACCCAA GCAGAAGCTT
E4	PA1396 fragment cloned into topology reporter plasmids *phoA* and *lacZ*	ATGAAGTTCGAG
N6	PA1396 fragment cloned into topology reporter plasmids *phoA* and *lacZ*	ATGAAGTTCGAGAAGAAT
V37	PA1396 fragment cloned into topology reporter plasmids *phoA* and *lacZ*	ATGAAGTTCGAGAAGAATACCGAGCTGGACCAGGCCA ACCTGCGACTGATCGTGGCCACCTGCGCGATCCTCTAC GTGGTGCTGATCGGCCTGCTGCCCGGCCTGAAGGTC
E38	PA1396 fragment cloned into topology reporter plasmids *phoA* and *lacZ*	ATGAAGTTCGAGAAGAATACCGAGCTGGACCAGGCCA ACCTGCGACTGATCGTGGCCACCTGCGCGATCCTCTAC GTGGTGCTGATCGGCCTGCTGCCCGGCCTGAAGGTCG AG
G66	PA1396 fragment cloned into topology reporter plasmids *phoA* and *lacZ*	ATGAAGTTCGAGAAGAATACCGAGCTGGACCAGGCCA ACCTGCGACTGATCGTGGCCACCTGCGCGATCCTCTAC GTGGTGCTGATCGGCCTGCTGCCCGGCCTGAAGGTCG AGACCTACCTGCCGATCGTTGCCTACTACGGCCTGTTC CTGATCGCCTCCATACTGCTGCGCCAGGCCATCGTGCG CTGGCCGGGG
A70	PA1396 fragment cloned into topology reporter plasmids *phoA* and *lacZ*	ATGAAGTTCGAGAAGAATACCGAGCTGGACCAGGCCA ACCTGCGACTGATCGTGGCCACCTGCGCGATCCTCTAC GTGGTGCTGATCGGCCTGCTGCCCGGCCTGAAGGTCG AGACCTACCTGCCGATCGTTGCCTACTACGGCCTGTTC CTGATCGCCTCCATACTGCTGCGCCAGGCCATCGTGCG CTGGCCGGGGCACTACCCGGCG
G107	PA1396 fragment cloned into topology reporter plasmids *phoA* and *lacZ*	ATGAAGTTCGAGAAGAATACCGAGCTGGACCAGGCCA ACCTGCGACTGATCGTGGCCACCTGCGCGATCCTCTAC GTGGTGCTGATCGGCCTGCTGCCCGGCCTGAAGGTCG AGACCTACCTGCCGATCGTTGCCTACTACGGCCTGTTC CTGATCGCCTCCATACTGCTGCGCCAGGCCATCGTGCG CTGGCCGGGGCACTACCCGGCGCGGCGGATCTTCTGC ATGCTGCACGACTAC GCCGGCACCTCGTTCGGCCTGATCGTGGGCGGCGAGG CAGCGCTGCCGCTGTACGCGGTGATGGTCTGGATCAA CCTGGGC
G109	PA1396 fragment cloned into topology reporter plasmids *phoA* and *lacZ*	ATGAAGTTCGAGAAGAATACCGAGCTGGACCAGGCCA ACCTGCGACTGATCGTGGCCACCTGCGCGATCCTCTAC GTGGTGCTGATCGGCCTGCTGCCCGGCCTGAAGGTCG AGACCTACCTGCCGATCGTTGCCTACTACGGCCTGTTC CTGATCGCCTCCATACTGCTGCGCCAGGCCATCGTGCG CTGGCCGGGGCACTACCCGGCGCGGCGGATCTTCTGC ATGCTGCACGACTACGCCGGCACCTCGTTCGGCCTGAT CGTGGGCGGCGAGGCAGCGCTGCCGCTGTACGCGGTG

		ATGGTCTGGATCAACCTGGGCAACGGC
A137	PA1396 fragment cloned into topology reporter plasmids *phoA* and *lacZ*	ATGAAGTTCGAGAAGAATACCGAGCTGGACCAGGCCA ACCTGCGACTGATCGTGGCCACCTGCGCGATCCTCTAC GTGGTGCTGATCGGCCTGCTGCCCGGCCTGAAGGTCG AGACCTACCTGCCGATCGTTGCCTACTACGGCCTGTTC CTGATCGCCTCCATACTGCTGCGCCAGGCCATCGTGCG CTGGCCGGGGCACTACCCGGCGCGGCGGATCTTCTGC ATGCTGCACGACTACGCCGGCACCTCGTTCGGCCTGAT CGTGGGCGGCGAGGCAGCGCTGCCGCTGTACGCGGTG ATGGTCTGGATCAACCTGGGCAACGGCATGCGCTACG GCTCGCGCTACCTGGCCATCGCCACCGCCCTGGCGCTG CTCGCGCTACTGGTCATCTATCGACTGACCCCGGCC
Q139	PA1396 fragment cloned into topology reporter plasmids *phoA* and *lacZ*	ATGAAGTTCGAGAAGAATACCGAGCTGGACCAGGCCA ACCTGCGACTGATCGTGGCCACCTGCGCGATCCTCTAC GTGGTGCTGATCGGCCTGCTGCCCGGCCTGAAGGTCG AGACCTACCTGCCGATCGTTGCCTACTACGGCCTGTTC CTGATCGCCTCCATACTGCTGCGCCAGGCCATCGTGCG CTGGCCGGGGCACTACCCGGCGCGGCGGATCTTCTGC ATGCTGCACGACTACGCCGGCACCTCGTTCGGCCTGAT CGTGGGCGGCGAGGCAGCGCTGCCGCTGTACGCGGTG ATGGTCTGGATCAACCTGGGCAACGGCATGCGCTACG GCTCGCGCTACCTGGCCATCGCCACCGCCCTGGCGCTG CTCGCGCTACTGGTCATCTATCGACTGACCCCGGCCTG GCAG
Q141	PA1396 fragment cloned into topology reporter plasmids *phoA* and *lacZ*	ATGAAGTTCGAGAAGAATACCGAGCTGGACCAGGCCA ACCTGCGACTGATCGTGGCCACCTGCGCGATCCTCTAC GTGGTGCTGATCGGCCTGCTGCCCGGCCTGAAGGTCG AGACCTACCTGCCGATCGTTGCCTACTACGGCCTGTTC CTGATCGCCTCCATACTGCTGCGCCAGGCCATCGTGCG CTGGCCGGGGCACTACCCGGCGCGGCGGATCTTCTGC ATGCTGCACGACTAC GCCGGCACCTCGTTCGGCCTGATCGTGGGCGGCGAGG CAGCGCTGCCGCTGTACGCGGTGATGGTCTGGATCAA CCTGGGCAACGGCATGCGCTACGGCTCGCGCTACCTG GCCATCGCCACCGCCCTGGCGCTGCTCGCGCTACTGGT CATCTATCGACTGACCCCGGCCTGGCAGGCTCAG
L205	PA1396 fragment cloned into topology reporter plasmids *phoA* and *lacZ*	ATGAAGTTCGAGAAGAATACCGAGCTGGACCAGGCCA ACCTGCGACTGATCGTGGCCACCTGCGCGATCCTCTAC GTGGTGCTGATCGGCCTGCTGCCCGGCCTGAAGGTCG AGACCTACCTGCCGATCGTTGCCTACTACGGCCTGTTC CTGATCGCCTCCATACTGCTGCGCCAGGCCATCGTGCG CTGGCCGGGGCACTACCCGGCGCGGCGGATCTTCTGC ATGCTGCACGACTACGCCGGCACCTCGTTCGGCCTGAT CGTGGGCGGCGAGGCAGCGCTGCCGCTGTACGCGGTG ATGGTCTGGATCAACCTGGGCAACGGCATGCGCTACG GCTCGCGCTACCTGGCCATCGCCACCGCCCTGGCGCTG CTCGCGCTACTGGTCATCTATCGACTGACCCCGGCCTG GCAGGCTCAGCCGTTCATGGTGCTGATGCTGATGACC ACCAGTACCGTCATTCCCTTCTACGCGCACCTCCTGCT GGAGCGCACGCGCAAGGCCACCGAGGAAGCGTTGCA GGCGAACCAGGAGAAATCGCGCCTGCTGGCCCAGGCC AGCCACGACCTGCGCCAGCCGATCCACTCCATCGGCC TGTTCACCGCCTGCCTG
T240	PA1396 fragment cloned into topology reporter plasmids *phoA* and *lacZ*	ATGAAGTTCGAGAAGAATACCGAGCTGGACCAGGCCA ACCTGCGACTGATCGTGGCCACCTGCGCGATCCTCTAC GTGGTGCTGATCGGCCTGCTGCCCGGCCTGAAGGTCG AGACCTACCTGCCGATCGTTGCCTACTACGGCCTGTTC CTGATCGCCTCCATACTGCTGCGCCAGGCCATCGTGCG CTGGCCGGGGCACTACCCGGCGCGGCGGATCTTCTGC ATGCTGCACGACTACGCCGGCACCTCGTTCGGCCTGAT CGTGGGCGGCGAGGCAGCGCTGCCGCTGTACGCGGTG ATGGTCTGGATCAACCTGGGCAACGGCATGCGCTACG GCTCGCGCTACCTGGCCATCGCCACCGCCCTGGCGCTG CTCGCGCTACTGGTCATCTATCGACTGACCCCGGCCTG GCAGGCTCAGCCGTTCATGGTGCTGATGCTGATGACC ACCAGTACCGTCATTCCCTTCTACGCGCACCTCCTGCT

		GGAGCGCACGCGCAAGGCCACCGAGGAAGCGTTGCA<br>GGCGAACCAGGAGAAATCGCGCCTGCTGGCCCAGGCC<br>AGCCACGACCTGCGCCAGCCGATCCACTCCATCGGCC<br>TGTTCACCGCCTGCCTGCGCGACGCCCGCCTGGGCGA<br>CGAGGAACGGCGCCTGGTGGACAACATCGACCGCTCG<br>CTGCTCAACGTCTCGCAACTGTTCCGCTCCATTCTCGA<br>TCTCTACACC
L300	PA1396 fragment cloned into topology reporter plasmids *phoA* and *lacZ*	ATGAAGTTCGAGAAGAATACCGAGCTGGACCAGGCCA<br>ACCTGCGACTGATCGTGGCCACCTGCGCGATCCTCTAC<br>GTGGTGCTGATCGGCCTGCTGCCCGGCCTGAAGGTCG<br>AGACCTACCTGCCGATCGTTGCCTACTACGGCCTGTTC<br>CTGATCGCCTCCATACTGCTGCGCCAGGCCATCGTGCG<br>CTGGCCGGGGCACTACCCGGCGCGGCGGATCTTCTGC<br>ATGCTGCACGACTACGCCGGCACCTCGTTCGGCCTGAT<br>CGTGGGCGGCGAGGCAGCGCTGCCGCTGTACGCGGTG<br>ATGGTCTGGATCAACCTGGGCAACGGCATGCGCTACG<br>GCTCGCGCTACCTGGCCATCGCCACCGCCCTGGCGCTG<br>CTCGCGCTACTGGTCATCTATCGACTGACCCCGGCCTG<br>GCAGGCTCAGCCGTTCATGGTGCTGATGCTGATGACC<br>ACCAGTACCGTCATTCCCTTCTACGCGCACCTCCTGCT<br>GGAGCGCACGCGCAAGGCCACCGAGGAAGCGTTGCA<br>GGCGAACCAGGAGAAATCGCGCCTGCTGGCCCAGGCC<br>AGCCACGACCTGCGCCAGCCGATCCACTCCATCGGCC<br>TGTTCACCGCCTGCCTGCGCGACGCCCGCCTGGGCGA<br>CGAGGAACGGCGCCTGGTGGACAACATCGACCGCTCG<br>CTGCTCAACGTCTCGCAACTGTTCCGCTCCATTCTCGA<br>TCTCTACACCCTCGACAACGGCCGGCTCCAACCCAAG<br>CAGGAGAACGTCCACCTGGGCGAGTTGCTGCGCGACC<br>TGGTCCGGCGCAACGCCGAAGCGGCGCGCTGGGCCGG<br>GGTGGAACTGCGCCTGCGCCCTTGCCGCCTGTGGACG<br>CGAACCGATCCGGGGCTGCTGTCGACCATGCTGCAGA<br>ACCTG
L381	PA1396 fragment cloned into topology reporter plasmids *phoA* and *lacZ*	ATGAAGTTCGAGAAGAATACCGAGCTGGACCAGGCCA<br>ACCTGCGACTGATCGTGGCCACCTGCGCGATCCTCTAC<br>GTGGTGCTGATCGGCCTGCTGCCCGGCCTGAAGGTCG<br>AGACCTACCTGCCGATCGTTGCCTACTACGGCCTGTTC<br>CTGATCGCCTCCATACTGCTGCGCCAGGCCATCGTGCG<br>CTGGCCGGGGCACTACCCGGCGCGGCGGATCTTCTGC<br>ATGCTGCACGACTACGCCGGCACCTCGTTCGGCCTGAT<br>CGTGGGCGGCGAGGCAGCGCTGCCGCTGTACGCGGTG<br>ATGGTCTGGATCAACCTGGGCAACGGCATGCGCTACG<br>GCTCGCGCTACCTGGCCATCGCCACCGCCCTGGCGCTG<br>CTCGCGCTACTGGTCATCTATCGACTGACCCCGGCCTG<br>GCAGGCTCAGCCGTTCATGGTGCTGATGCTGATGACC<br>ACCAGTACCGTCATTCCCTTCTACGCGCACCTCCTGCT<br>GGAGCGCACGCGCAAGGCCACCGAGGAAGCGTTGCA<br>GGCGAACCAGGAGAAATCGCGCCTGCTGGCCCAGGCC<br>AGCCACGACCTGCGCCAGCCGATCCACTCCATCGGCC<br>TGTTCACCGCCTGCCTGCGCGACGCCCGCCTGGGCGA<br>CGAGGAACGGCGCCTGGTGGACAACATCGACCGCTCG<br>CTGCTCAACGTCTCGCAACTGTTCCGCTCCATTCTCGA<br>TCTCTACACCCTCGACAACGGCCGGCTCCAACCCAAG<br>CAGGAGAACGTCCACCTGGGCGAGTTGCTGCGCGACC<br>TGGTCCGGCGCAACGCCGAAGCGGCGCGCTGGGCCGG<br>GGTGGAACTGCGCCTGCGCCCTTGCCGCCTGTGGACG<br>CGAACCGATCCGGGGCTGCTGTCGACCATGCTGCAGA<br>ACCTGCTCTCCAACAGCTTCAAGTACGCCGCCGAGCG<br>CCCGCTGCTGATCGGCGTGCGGCGGCGAGGCGACGGC<br>CTGGCCGTAGCCATCTACGACCAGGGCCGGGGGATCG<br>CGGAGGAACACCTGCCGCGGGTGTTCGAGGAGTTCTA<br>CCGGGTACGCGAGACGCGCGACCGCGACGTCGAGGG<br>AATCGGCCTGGGGCTGTCCATCGTCCGCCGCCTGGGG<br>CAGTTGACCGGGATCGAGGTGACGCTG

Supplementary Note 1

Experimental Procedures and Spectroscopic Data for Compounds

All synthetic procedures and analytical data for compounds used in this study are detailed below or the ChEBI identifiers are provided. Compounds not synthesized were purchased from Sigma-Aldrich, Cayman Chemical, Chemieliva Pharmaceutical Co., Ltd, New Chem, SynCom and HEOWNS.

Preparation of BDSF

Decanal

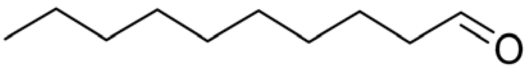

Synthetic scheme:

A solution of anhydrous DMSO (1.44 mL, 20.22 mmoL) in anhydrous CH_2Cl_2 (25 mL) was treated with oxalyl chloride (0.87 mL, 10.11 mmoL) and stirred for 30 minutes at -78°C. Decanol (1.2 mL, 6.30 mmoL) was added and the reaction mixture stirred for 1.5 h at -78°C. Triethylamine (4.23 mL, 30.33 mmoL) was added and the reaction warmed to rt. The reaction mixture was re-cooled to 0°C, $NH_4Cl_{(sat.)}$ (10mL) added and partitioned between CH_2Cl_2 (25 mL). The aqueous layer was re-extracted with CH_2Cl_2 (2 x 25 mL), dried over $MgSO_4$ and the solvent removed under reduced pressure. Purification by column chromatography on silica gel eluting with Hex:EtOAc (96:4) gave the product as a colourless oil (0.863 g, 87 %).

^{1}H NMR (400 MHz, $CDCl_3$) δ: 9.76 (s, 1H, CHO), 2.41 (td, 2H, J = 15.4, 9.2, 1.9 Hz) 1.66 - 1.58 (m, 2H), 1.33 -1.22 (m, 12H), 0.86 (t, 3H, J = 7.0 Hz).

^{13}C NMR (75 MHz, $CDCl_3$): δ 202.5, 43.8, 31.8, 29.3, 29.3, 29.2, 29.1, 22.6, 22.0, 13.9.

IR (NaCl disk): 3330.38, 2926.66 cm^{-1}.

Ethyl (*Z*)-dodec-2-enoate and Ethyl (*E*)-dodec-2-enoate

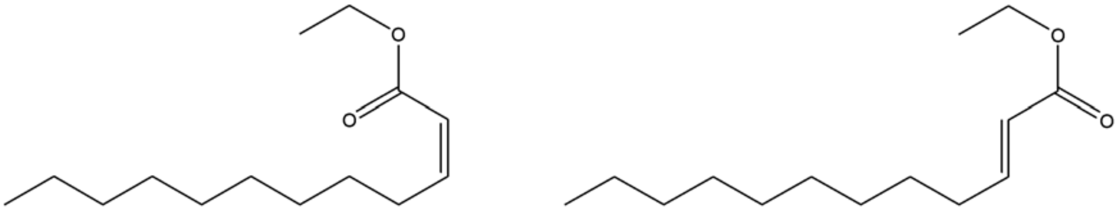

Synthetic scheme:

To a solution of sodium hydride (95%) (451 mg, 17.9 mmoL) in anhydrous THF (130 mL) was added ethyl[bis(2,2,2-trifluoroethoxy)phosphyl]acetate (2.13 mL, 8.95 mmoL) which was stirred for 45 minutes at 0°C. Decanal (1.39 g, 8.91 mmoL) was added dropwise and stirred for 30 minutes at -78°C. H_2O (20 mL) was added and the reaction stirred for 30 minutes at rt. The solvent was removed under reduce pressure and partitioned between EtOAc (40 mL) and H_2O (40 mL). The organic layer was re-extracted with EtOAc (2 x 40 mL) and washed with brine (40 mL). The combined organic layers were dried over $MgSO_4$ and evaporated to dryness to yield the crude product as colourless oil. Purification using preparative thin layer chromatography eluting with Hex:EtOAc (96:4) gave the product as a mixture of the *Z*-isomer (1.29 g, 64%) and the *E*-isomer (0.211 g, 10%)

Ethyl (*Z*)-dodec-2-enoate:

^{1}H NMR (400 MHz, $CDCl_3$) δ: (**5a**) 6.21 (td, 1H, *J* = 11.5, 7.5 Hz), 5.75 (td, 1H, *J* = 12.3, 1.7 Hz), 4.16 (q, 2H, *J* = 7.1 Hz), 2.64 (dt, 2H, *J* = 14.8, 5.7, 1.6 Hz), 1.46 – 1.38 (m, 2H), 1.43-1.31 (m, 15H), 0.87 (t, 3H, *J* = 6.6 Hz).

^{13}C NMR (125 MHz, $CDCl_3$) δ: (**5a**) 166.5, 150.6, 119.5, 59.7, 31.8, 29.5, 29.4, 29.3, 29.3, 29.0, 29.0, 22.6, 14.2, 14.11.

HRMS $[M+H]^+$: 227.2011, $C_{14}H_{27}O_2^+$ requires, 227.2012.

ES-MS: *m/z* 227.3 $[M+H]^+$, $C_{14}H_{27}O_2^+$.

IR (NaCl disk): 2926, 1723, 1183 cm^{-1}

Ethyl (*E*)-dodec-2-enoate:

^{1}H NMR (400 MHz, $CDCl_3$) δ: 6.95 (td, 1H, *J* = 15.5, 7.0 Hz), 5.79 (td, 1H, *J* = 15.7, 1.52 Hz), 4.17 (q, 2H, *J* = 7.1 Hz), 2.18 (dq, 3H, *J* = 14.5, 7.0, 1.5 Hz), 1.47 – 1.40 (m, 2H), 1.33 – 1.23 (m, 15H), 0.86 (t, 3H, *J* = 6.6 Hz).

^{13}C NMR (125 MHz, $CDCl_3$) δ: 166.8, 149.5, 121.1, 60.1, 32.2, 31.9, 29.5, 29.4, 29.3, 29.1, 28.0, 22.6, 14.2, 14.1.

HRMS $[M+H]^+$: 227.2011, $C_{14}H_{27}O_2^+$ requires, 227.2000.

ES-MS: *m/z* 227.3 [M+H]+, $C_{14}H_{27}O_2^+$.

C2: (*Z*)-2-Dodecenoic acid (BDSF)

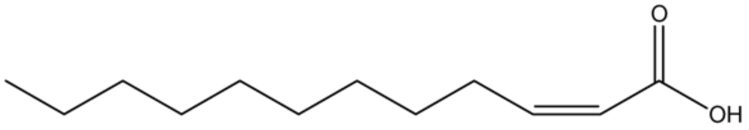

Synthetic scheme:

A solution of ethyl (*Z*)-dodec-2-enoate (0.327 g, 1.44 mmoL) in THF:MeOH (2:1) (4 mL) was treated with lithium hydroxide (0.332 g, 14.45 mmoL) in H_2O (2.5 mL) 0°C and then stirred at rt for 24 h. The reaction mixture was cooled to 0°C, H_2O (10 mL) added and the pH was adjusted to 1. The solvent was removed under reduced pressure and partitioned between CH_2Cl_2 (20 mL) and H_2O (20 mL). The aqueous layer was re-extracted with CH_2Cl_2 (2 x 20 mL), and the combined organics dried over $MgSO_4$ and evaporated to dryness. Purification *via* column chromatography on silica gel eluting with Hex:EtOAc (85:15) gave the product as a colourless oil 0.28 g, 98 %.

^{1}H NMR (400 MHz, $CDCl_3$) δ: 11.64 (bs, 1H), 6.35 (dt, 1H, J = 11.4, 7.6 Hz), 5.77 (dt, 1H, J = 11.5, 1.6 Hz), 2.65 (dt, 2H, J = 14.8, 7.5, 1.96 Hz), 1.47 – 1.40 (m, 2H), 1.34-1.26 (m, 12H), 0.88 (t, 3H, J = 6.7 Hz).

^{13}C NMR (125 MHz, $CDCl_3$) δ: 171.9, 153.5, 118.9, 31.8, 29.7, 29.5, 29.4, 29.2, 29.2, 28.9, 22.6, 14.08.

HRMS $[M+H]^+$: 199.1698, $C_{12}H_{23}O_2^{+,}$ requires 199.1706.

ES-MS: *m/z* 199.6 $[M+H]^+$, $C_{12}H_{23}O_2^+$.

IR (NaCl disk): 2926, 1698, 1456, 1241 cm^{-1}.

ChEBI identifier: 38372

C15: (*E*)-Dodec-2-enoic acid (*trans*-BDSF)

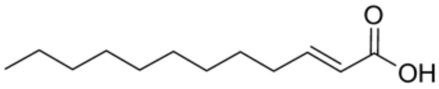

Synthetic scheme:

A solution of ethyl (*E*)-dodec-2-enoate (80 mg, 0.35 mmoL) in THF:MeOH (2:1) (3 mL) was treated with lithium hydroxide (84 mg, 3.53 mmoL) in H_2O (1 mL) at 0°C and then stirred for 24 h at rt. The reaction mixture was cooled to 0°C, H_2O (10 mL) added and acidified to pH 1. The solvent was removed under reduced pressure and partitioned between CH_2Cl_2 (20 mL) and H_2O (20 mL). The aqueous layer was re-extracted with CH_2Cl_2 (2 x 20 mL), and the combined organics dried over $MgSO_4$ and evaporated to dryness. Purification *via* silica gel eluting with Hex:EtOAc (85:15) gave the product as a colourless oil 59 mg, 85 %.

^{1}H NMR (400 MHz, $CDCl_3$) δ: 11.2 (bs, 1H), 7.08 (dt. 1H, *J* = 19.8, 7 Hz), 5.84-5.79 (m, 1H), 2.25 – 2.19 (m, 2H), 1.50 – 1.41 (m, 2H), 1.34 – 1.21 (m, 12H), 0.87 (t, 3H, *J* = 6.6 Hz).

HRMS $[M+H]^+$: , $C_{12}H_{23}O_2^{+,}$ requires 199.0276.

ES-MS: *m/z* 199.3 $[M+H]^+$, $C_{13}H_{22}O_2^+$.

Preparation of DSF

9-Methyldecan-1-ol

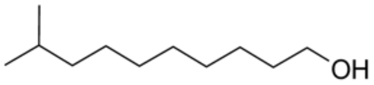

Synthetic scheme:

A solution of Mg turnings (403 mg, 16.57 mmoL) and *i*-pentyl bromide (2.5 g, 16.57 mmoL) were stirred in anhydrous THF (7 mL). The mixture was treated with solution of 6-bromohexan-1-ol (0.722 mL, 5.52 mmoL) and Li_2CuCl_4 (0.1 M, 2.1 mL in THF) and stirred for 1 h at rt. $HCl_{(conc.)}$ (5 mL) and Et_2O (10 mL) were added. The sticky solid was washed with Et_2O (3 x 10 mL), stirred and decanted. The organic layer was washed with $NaHCO_3$ (30 mL) and brine (30 mL), dried over $MgSO_4$ and the solvent removed under reduced pressure. Purification by column chromatography on silica gel eluting with Hex:EtOAc (2:1) gave the product as a colourless oil (0.505 g, 53 %).

^{1}H NMR (400 MHz, $CDCl_3$) δ: 3.63 (t, 2H, J = 6.6 Hz), 1.62 - 1.46 (m, 4H), 1.36 - 1.20 (m, 9H), 1.17 – 1.12 (m, 2H), 0.86 (t, 6H, J = 6.6 Hz).

^{13}C NMR (125 MHz, $CDCl_3$) δ: 63.1, 39.0, 32.8, 29.8, 29.6, 29.4, 27.9, 27.3, 25.7, 22.6.

ES-MS: *m/z* 171.4 $[M-H]^-$, $C_{11}H_{23}O^-$.

IR (NaCl disk): 3440, 2925 cm^{-1}.

ChEBI identifier: 87138

9-Methyldecanal

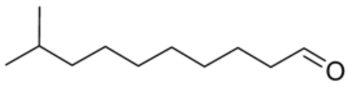

Synthetic scheme:

A solution of anhydrous DMSO (1.24 mL, 17.53 mmoL) in anhydrous CH_2Cl_2 (25 mL) was treated with oxalyl chloride (0.75 mL, 8.78 mmoL) and stirred for 30 minutes at -78°C. 9-Methyldecan-1-ol (0.95 g, 5.5 mmoL) was added and the reaction mixture stirred for 1.5 h at -78°C. Triethylamine (3.7 mL, 26.3 mmoL) was added and the reaction warmed to rt. The reaction mixture was re-cooled to 0°C, $NH_4Cl_{(sat.)}$ (10 mL) added and partitioned with CH_2Cl_2 (25 mL). The aqueous layer was re-extracted with CH_2Cl_2 (2 x 25 mL), dried over $MgSO_4$ and the solvent removed under reduced pressure. Purification by column chromatography on silica gel eluting with Hex:EtOAc (96:4) gave the product as a colourless oil (0.691 g, 74 %).

^{1}H NMR (400 MHz, $CDCl_3$) δ: 9.76 (s, 1H, CHO), 2.42 (dt, 2H, J = 9.2, 7.4, 1.8 Hz), 1.63 (q, 2H, J = 7.3, 14.6 Hz), 1.46 – 1.56 (m, 1H), 1.34 -1.26 (m, 8H), 1.17 - 1.12 (m, 2H), 0.86 (d, 6H, J = 6.6 Hz).

^{13}C NMR (125 MHz, $CDCl_3$) δ: 202.9, 43.9, 38.9, 29.6, 29.3, 29.1, 27.9, 27.3, 22.6, 22.0.

IR (NaCl disk): 2927, 1729 cm^{-1}.

C26: Ethyl (*Z*)-11-methyldodec-2-enoate and Ethyl (*E*)-11-methyldodec-2-enoate

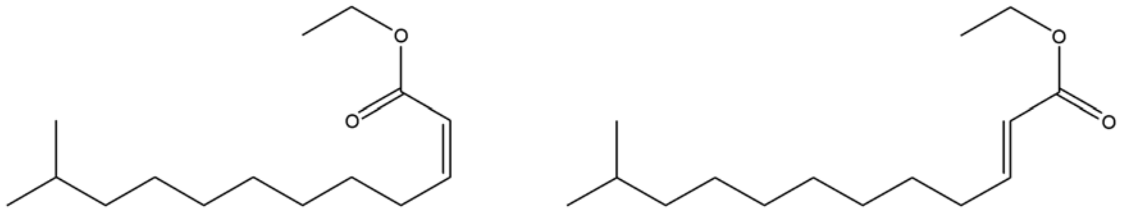

Synthetic scheme:

To a solution of sodium hydride (95%) (204 mg, 8.1 mmoL) in anhydrous THF (60 mL) was added ethyl[bis(2,2,2-trifluoroethoxy)phosphinyl]acetate (962 µL, 4.05 mmoL) which was stirred at 0°C for 45 minutes. 9-Methyldecanal (0.69 g, 4.05 mmoL) was added drop-wise and stirred for 30 minutes at -78°C. H_2O (50 mL) was added and the reaction stirred for at rt 30 minutes. The solvent was removed under reduced pressure and partitioned between EtOAc (60 mL) and H_2O (60 mL). The organic layer was re-extracted with EtOAc (2 x 60 mL) and washed with brine (60 mL). The combined organic layers were dried over $MgSO_4$ and evaporated to dryness to yield the crude product as colourless oil. Purification using preparative thin layer chromatography eluting with Hex:EtOAc (96:4) gave the product as a mixture of the *Z*-isomer (0.472 g, 48%) and *E*-isomer (0.173 g, 17%)

Ethyl (*Z*)-11-methyldodec-2-enoate

^{1}H NMR (400 MHz, $CDCl_3$) δ: 6.21 (td, 1H, *J* = 11.5, 7.6 Hz), 5.75 (td, 1H, *J* = 11.5, 1.5 Hz), 4.16 (q, 2H, *J* = 7.2 Hz), 2.64 (dq, 2H, *J* = 14.9, 7.3, 1.5 Hz), 1.34 – 1.58 (m, 3H), 1.31 – 1.25 (m, 11H), 1.16 - 1.12 (m, 2H), 0.85 (d, 6H, *J* = 6.6 Hz).

^{13}C NMR (75 MHz, $CDCl_3$) δ: 166.5, 150.7, 119.5, 59.7, 39.0, 29.8, 29.4, 29.3, 29.0, 29.0, 27.9, 27.3, 22.6, 14.2.

HRMS $[M+H]^+$: 241.2168, $C_{15}H_{29}O_2^+$ requires, 241.2172.

ES-MS: *m/z* 241.3 [M+H]+, $C_{15}H_{29}O_2^+$.

IR (NaCl disk): 2926, 1723, 1183 cm^{-1}.

Ethyl (*E*)-11-methyldodec-2-enoate

^{1}H NMR (400 MHz, $CDCl_3$) δ: 6.96 (td, 1H, *J* = 15.6, 6.9 Hz), 5.80 (td, 1H, *J* = 15.6, 1.5 Hz), 4.18 (q, 2H, *J* = 7.1 Hz), 2.27 (dq, 2H, *J* = 12.8, 7.5, 1.4 Hz), 1.54 – 1.41 (m, 3H), 1.33 – 1.24 (m, 11H), 1.16 - 1.11 (m, 2H), 0.85 (d, 6H, *J* = 6.6 Hz).

^{13}C NMR (75 MHz, $CDCl_3$) δ: 166.8, 149.5, 121.1, 60.1, 39.0, 32.2, 29.7, 29.4, 29.1, 28.0, 27.9, 27.3, 22.6, 14.2.

HRMS $[M+H]^+$: 241.2158, $C_{15}H_{29}O_2^+$ requires, 241.2168.

ES-MS: *m/z* 241.3 $[M+H]^+$, $C_{15}H_{29}O_2^+$.

C1: *(Z)*-11-Methyl-2-dodecenoic acid (DSF)

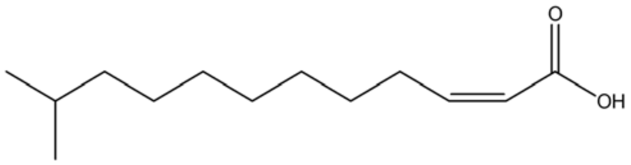

Synthetic scheme:

A solution of ethyl (*Z*)-11-methyldodec-2-enoate (377 mg, 1.57 mmoL) in THF:MeOH (2:1) (10 mL) was treated with lithium hydroxide (306 mg, 15.65 mmoL) in H_2O (3 mL) 0°C and then stirred at rt for 24 h. The reaction mixture was cooled to 0°C, H_2O (10 mL) added and the pH was adjusted to 1. The solvent was removed under reduced pressure and partitioned between CH_2Cl_2 (20 mL) and H_2O (20 mL). The aqueous layer was re-extracted with CH_2Cl_2 (2 x 20 mL), and the combined organics dried over $MgSO_4$ and evaporated to dryness. Purification by silica gel chromatography with Hex:EtOAc (85:15) as eluant gave the product as a colourless oil 292 mg, 88 %.

^{1}H NMR (400 MHz, $CDCl_3$) δ: 12.0 (bs, 1H), 6.35 (td, 1H, *J* = 11.5, 7.0 Hz), 5.79 (td, 1H, *J* = 11.5, 1.7 Hz) 2.66 (dq, 2H, *J* = 7.44, 1.7 Hz), 1.56 - 1.41 (m, 3H), 1.36 -1.22 (m, 8H), 1.17 - 1.12 (m, 2H), 0.86 (d, 6H, *J* = 6.6 Hz).

^{13}C NMR (125 MHz, $CDCl_3$) δ: 171.2, 153.6, 118.7, 39.0, 29.8, 29.4, 29.2, 29.2, 28.9, 27.9, 27.3, 22.6.

HRMS $[M+H]^+$: 213.1855, $C_{13}H_{25}O_2^+$ requires, 213.1854.

ES-MS: *m/z* 213.1 $[M+H]^+$, $C_{13}H_{25}O_2^+$.

IR (NaCl disk): 2926, 1697, 1436, 1242 cm^{-1}.

ChEBI identifier: 81585

C14: *(E)*-11-Methyldodec-2-enoic acid (*trans*-DSF)

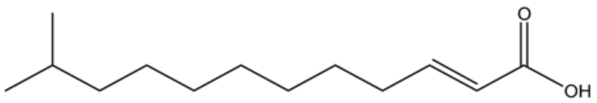

Synthetic scheme:

A solution of ethyl (*Z*)-11-methyldodec-2-enoate (0.127 g, 0.53 mmoL) in THF:MeOH (2:1) (4 mL) was treated with lithium hydroxide (0.145, 6.26 mmoL) in H_2O (2 mL) at 0°C and then stirred at rt for 24 h. The reaction mixture was cooled to 0°C, H_2O (10 mL) added and adjusted to pH 1. The solvent was removed under reduced pressure and partitioned between CH_2Cl_2 (20 mL) and H_2O (20 mL). The aqueous layer was re-extracted with CH_2Cl_2 (2 x 20 mL), and the combined organics dried over $MgSO_4$ and evaporated to dryness. Purification *via* column chromatography on silica fel eluting with Hex:EtOAc (85:15) gave the product a colourless oil 0.107 g, 95 %.

^{1}H NMR (400 MHz, $CDCl_3$) δ: 11.2 (bs, 1H), 7.12 – 7.05, (m, 1H), 5.84 – 5.80 (m, 1H), 2.25 – 2.20 (m, 2H), 1.56 – 1.42 (m, 3H), 1.32 - 1.23 (m, 8H), 1.17-1.11 (m, 2H), 0.86 (d, 6H, J = 5.7 Hz).

^{13}C NMR (125 MHz, $CDCl_3$) δ: 172.1, 152.5, 120.5, 39.0, 32.3, 29.7, 29.4, 29.2, 29.1, 27.9, 27.8, 27.3, 22.6.

HRMS $[M+H]^+$: 213.1855, $C_{13}H_{25}O_2^+$ requires, 213.1854.

ES-MS: *m/z* 213.4 $[M+H]^+$, $C_{13}H_{25}O_2^+$.

Preparation of DSF analogues

C23: *(Z)*-11-Methyl-*N*-(methylsulfonyl)dodec-2-enamide

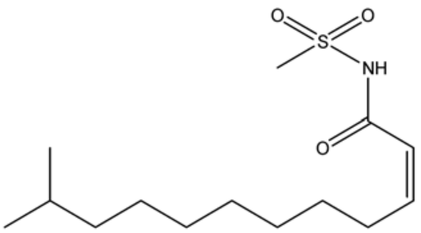

Synthetic scheme:

A solution of (*Z*)-11-methyl-2-dodecenoic acid (DSF) (27.8 mg, 0.131 mmol) in anhydrous DCM (5 ml) at 0ºC was treated with dimethylaminopyridine (4.9 mg, 0.04 mmol), EDCI (34.9 mg, 0.183 mmol) and methanesulfonamide (35.9 mg, 0.378 mmol) and then stirred at room temperature for 24 hours. Saturated $NaHCO_3$ solution (10 ml) was added. The organic layer was separated and volatiles evaporated. Initial purification by silica chromatography (0-10% MeOH in DCM), followed by reverse phase HPLC (5-95% MeCN in 0.1% NH_4OH) yielded the desired product as a colourless oil (0.5 mg, 0.001 mmol, 1.2%).

^{1}H NMR (500 MHz, $CDCl_3$) δ: 7.08 - 7.06 (1H, m), 5.81 (1H, d, J=15.3 Hz), 3.33 (3H, s), 2.24 (2H, q, J=6.7 Hz), 1.52 (2H, m), 1.47 (2H, m), 1.28 (9H, dd, J=4.1, 18.8 Hz), 1.18 - 1.14 (2H, m), 0.87 (6H, d, J=6.6 Hz);

ES-MS: *m/z* 290.1 [M+H]+, $C_{14}H_{28}NO_3S^+$.

ChEBI identifier: 87144

C24: *(Z)*-*N*-Cyclopropyl-11-methyldodec-2-enamide

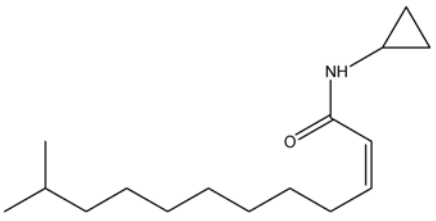

A solution of (*Z*)-11-methyl-2-dodecenoic acid (DSF) (25mg, 0.117 mmol) in anhydrous DCM (5 ml) at 20ºC was treated with cyclopropylamine (24.5 uL. 0.353 mmol), DIPEA (41 uL, 0.235 mmol) and propylphosphonic anhydride solution (50% in EtOAc, 2.4 ml) then stirred at room temperature for 24 hours. Saturated $NaHCO_3$ solution (10 ml) was added. The organic layer was separated and volatiles evaporated. Initial purification by silica chromatography (0-10% MeOH in DCM), followed by reverse phase HPLC (5-95% MeCN in 0.1% NH_4OH) yielded the desired product as a colourless oil (14.4 mg, 0.054 mmol, 46%).

^1H NMR (500 MHz, $CDCl_3$) δ: 6.01 - 5.95 (1H, m), 5.63 - 5.54 (2H, m), 2.78 - 2.73 (1H, m), 2.68 - 2.62 (2H, m), 1.54 - 1.40 (1H, m), 1.32 - 1.23 (8H, m), 1.15 (2H, q, J=6.9 Hz), 0.87 - 0.85 (10H, m), 0.54 - 0.50 (2H, m);.

^{13}C NMR (125 MHz, $CDCl_3$) δ: 168.0, 146.3, 121.7, 39.1, 29.8, 29.5, 29.4, 28.8, 28.0, 27.4, 22.7, 22.4, 6.7.

ES-MS: *m/z* 252.2 [M+H]+, $C_{16}H_{30}NO^+$.

ChEBI identifier: 87143

Other analogues

C3: *(Z)*-10-Methyldodec-2-enoic acid

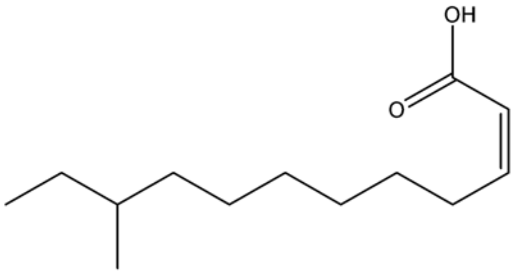

PubChem CID: 129730386

C4: *(Z)*-9-Methyldodec-2-enoic acid

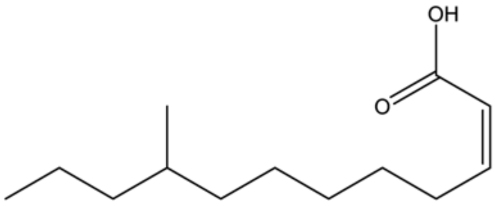

C5: *(Z)*-8-Methyldodec-2-enoic acid

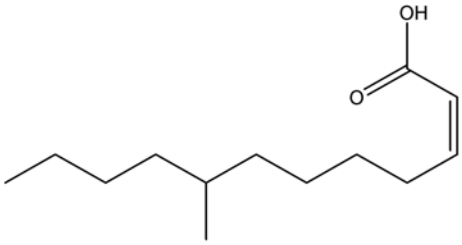

C6: *(Z)*-7-Methyldodec-2-enoic acid

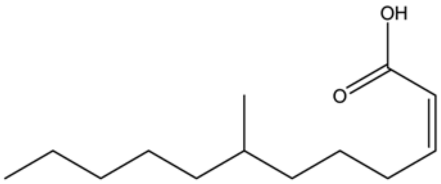

C7: *(Z)*-6-Methyldodec-2-enoic acid

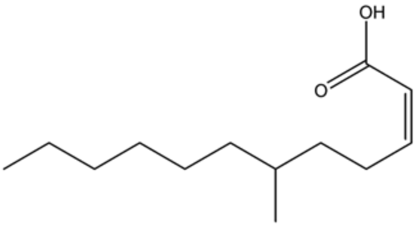

C8: *(Z)*-12-Methyltridec-2-enoic acid

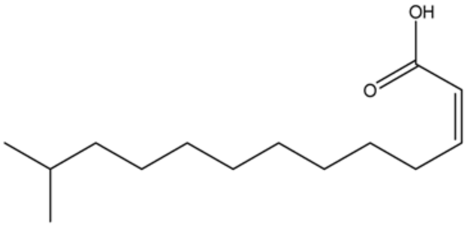

C9: *(Z)*-Tridec-2-enoic acid

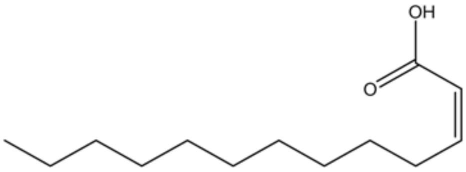

PubChem CID: 5356766

C10: *(Z)*-13-Methyltetradec-2-enoic acid

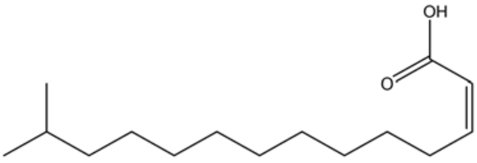

ChEBI identifier: 87148

C11: *(Z)*-Tetradec-2-enoic acid

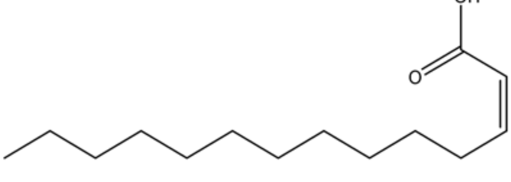

PubChem identifier: 5362743

C12: *(Z)*-14-Methylpentadec-2-enoic acid

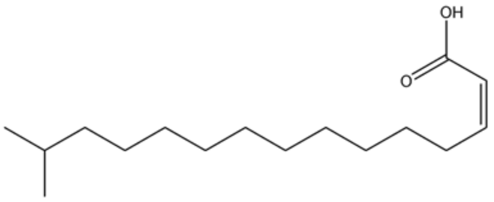

CHEBI:87146

C13: *(Z)*-Pentadec-2-enoic acid

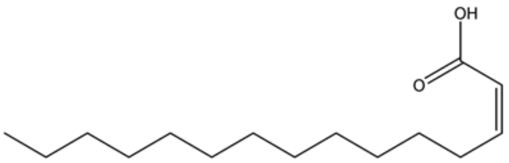

PubChem CID: 53887649

C15: *(E)*-Dodec-2-enoic acid

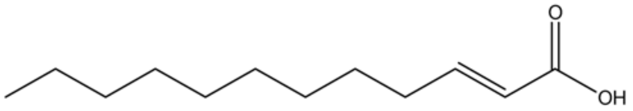

ChEBI identifier: 37162

C16: *(E)*-Undec-2-enoic acid

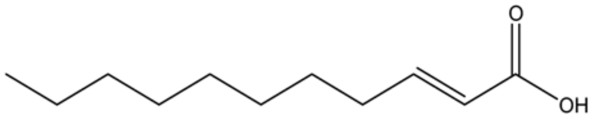

ChEBI identifier: 39450

C17: *(E)*-10-Methylundec-2-enoic acid

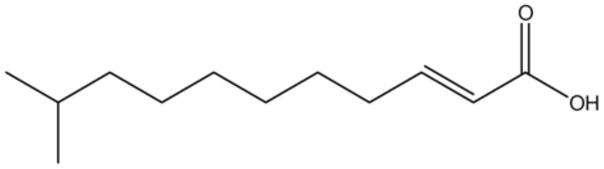

PubChem identifier: 53804867

C18: (2*Z*, 4*E*)-11-Methyldodeca-2,4-dienoic acid

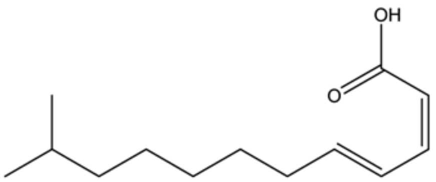

PDB: 0W5

C19: (2*Z*,4*E*)-Dodeca-2,4-dienoic acid

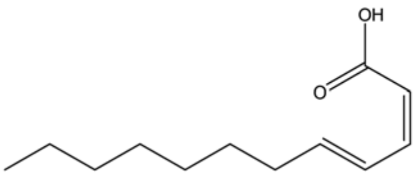

C20: (2*E*, 4*E*)-10-Methylundeca-2,4-dienoic acid

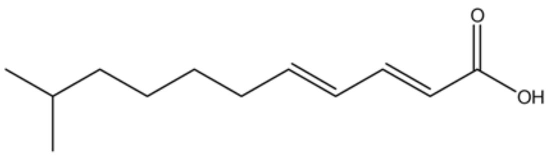

C21: (2*E*,4*E*)-Undeca-2,4-dienoic acid

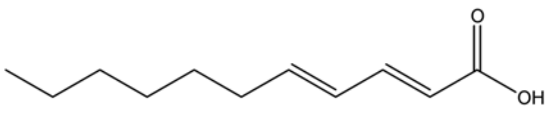

PubChem CID: 5312374

C22: *(Z)*-*N*-hydroxy-11-methyldodec-2-enamide

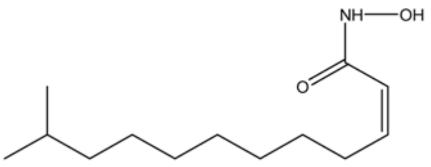

ChEBI identifier: 87145

C25: *(Z)*-Methyl 11-methyldodec-2-enoate

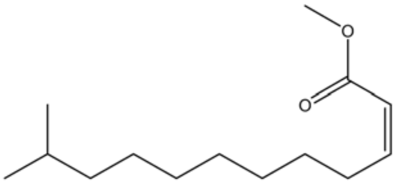

ChEBI identifier: 87151

C27: 6-(6-Methylheptyl)pyridin-2-amine

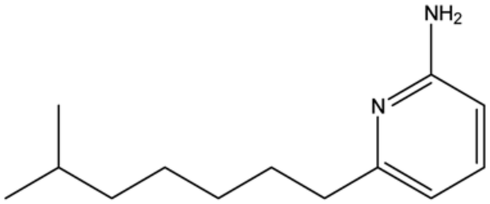

C28: *N*-Cyclopropyl-6-(6-methylheptyl)pyridin-2-amine

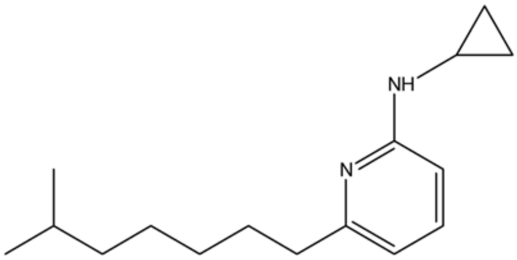

CHEBI: 38785

Supplementary References

1. Ryan, R. P. et al. Interspecies signalling via the Stenotrophomonas maltophilia diffusible signal factor influences biofilm formation and polymyxin tolerance in Pseudomonas aeruginosa. Mol. Microbiol. (2008). doi:10.1111/j.1365-2958.2008.06132.x
2. Twomey, K. B. et al. Bacterial cis-2-unsaturated fatty acids found in the cystic fibrosis airway modulate virulence and persistence of Pseudomonas aeruginosa. ISME J. (2012). doi:10.1038/ismej.2011.167
3. Chew, S. C. et al. Matrix Polysaccharides and SiaD Diguanylate Cyclase Alter Community Structure and Competitiveness of Pseudomonas aeruginosa during Dual-Species Biofilm Development with Staphylococcus aureus. MBio 9, e00585-18 (2018).
4. Haagensen, J. A. J. et al. Differentiation and distribution of colistin- and sodium dodecyl sulfate-tolerant cells in Pseudomonas aeruginosa biofilms. J. Bacteriol. (2007). doi:10.1128/JB.00720-06
5. Cunneen, M. M. & Reeves, P. R. Membrane topology of the Salmonella enterica serovar Typhimurium Group B O-antigen translocase Wzx. FEMS Microbiol. Lett. (2008). doi:10.1111/j.1574-6968.2008.01295.x
6. An, S. Q. et al. High-resolution transcriptional analysis of the regulatory influence of cell-to-cell signalling reveals novel genes that contribute to Xanthomonas phytopathogenesis. Mol. Microbiol. (2013). doi:10.1111/mmi.12229
