# Supplementary material for: Modulation of antibiotic sensitivity and biofilm formation in Pseudomonas aeruginosa by interspecies signal analogues
Source: Nat Commun. 2019 May 27;10:2334. doi: 10.1038/s41467-019-10271-4 (PMC6536496; doi:10.1038/s41467-019-10271-4)
Supplement: Supplementary file 2 — Reporting Summary [file 41467_2019_10271_MOESM2_ESM.pdf]

nature research

Corresponding author(s): Shi-qi An
Miguel A. Valvano
Ji-liang Tang

Last updated by author(s): Mar 29, 2019

Reporting Summary

Nature Research wishes to improve the reproducibility of the work that we publish. This form provides structure for consistency and transparency in reporting. For further information on Nature Research policies, see Authors & Referees and the Editorial Policy Checklist.

Statistics

For all statistical analyses, confirm that the following items are present in the figure legend, table legend, main text, or Methods section.

n/a	Confirmed	
☐	☒	The exact sample size (*n*) for each experimental group/condition, given as a discrete number and unit of measurement
☒	☐	A statement on whether measurements were taken from distinct samples or whether the same sample was measured repeatedly
☐	☒	The statistical test(s) used AND whether they are one- or two-sided<br>*Only common tests should be described solely by name; describe more complex techniques in the Methods section.*
☒	☐	A description of all covariates tested
☐	☒	A description of any assumptions or corrections, such as tests of normality and adjustment for multiple comparisons
☐	☒	A full description of the statistical parameters including central tendency (e.g. means) or other basic estimates (e.g. regression coefficient) AND variation (e.g. standard deviation) or associated estimates of uncertainty (e.g. confidence intervals)
☒	☐	For null hypothesis testing, the test statistic (e.g. *F*, *t*, *r*) with confidence intervals, effect sizes, degrees of freedom and *P* value noted<br>*Give P values as exact values whenever suitable.*
☒	☐	For Bayesian analysis, information on the choice of priors and Markov chain Monte Carlo settings
☒	☐	For hierarchical and complex designs, identification of the appropriate level for tests and full reporting of outcomes
☒	☐	Estimates of effect sizes (e.g. Cohen's *d*, Pearson's *r*), indicating how they were calculated

Our web collection on statistics for biologists contains articles on many of the points above.

Software and code

Policy information about availability of computer code

Data collection	CLC Biomedical Genomics Workbench 4.0
Data analysis	CLC Biomedical Genomics Workbench 4.0

For manuscripts utilizing custom algorithms or software that are central to the research but not yet described in published literature, software must be made available to editors/reviewers. We strongly encourage code deposition in a community repository (e.g. GitHub). See the Nature Research guidelines for submitting code & software for further information.

Data

Policy information about availability of data

All manuscripts must include a data availability statement. This statement should provide the following information, where applicable:

- Accession codes, unique identifiers, or web links for publicly available datasets
- A list of figures that have associated raw data
- A description of any restrictions on data availability

Microarray source data that support the findings of this study have been deposited in Gene Expression Omnibus (GEO) database with the accession codes GSE110126.
The data underlying Figs 1c, 1d, 2b, 2c, 3a, 3b, 3d, 4a, 4b, 4c, 4e, 5a, 5b, 5c and Supplementary Figs 1-12 and Tables 1-2 are provided as a Source Data

Field-specific reporting

Please select the one below that is the best fit for your research. If you are not sure, read the appropriate sections before making your selection.

☒ Life sciences ☐ Behavioural & social sciences ☐ Ecological, evolutionary & environmental sciences

For a reference copy of the document with all sections, see nature.com/documents/nr-reporting-summary-flat.pdf

Life sciences study design

All studies must disclose on these points even when the disclosure is negative.

Sample size	N.A.
Data exclusions	N.A.
Replication	N.A.
Randomization	N.A.
Blinding	N.A.

Reporting for specific materials, systems and methods

We require information from authors about some types of materials, experimental systems and methods used in many studies. Here, indicate whether each material, system or method listed is relevant to your study. If you are not sure if a list item applies to your research, read the appropriate section before selecting a response.

Materials & experimental systems

n/a	Involved in the study	
☐	☒	Antibodies
☐	☒	Eukaryotic cell lines
☒	☐	Palaeontology
☐	☒	Animals and other organisms
☒	☐	Human research participants
☒	☐	Clinical data

Methods

n/a	Involved in the study	
☒	☐	ChIP-seq
☒	☐	Flow cytometry
☒	☐	MRI-based neuroimaging

Antibodies

Antibodies used	Anti-6X His tag antibody (HRP) Abcam , Anti-Mycm (Invitrogen)
Validation	Please see manufacture websites.

Eukaryotic cell lines

Policy information about cell lines

Cell line source(s)	CFBE41-cells(CFBE cells) are human bronchial epithelial cells (Bruce Stanton & George O'Toole lab)
Authentication	Standard phenotype/Molecular biology screen
Mycoplasma contamination	Negative for MYcoplasma. PCR and phenotype negative.
Commonly misidentified lines (See ICLAC register)	N.A.

Animals and other organisms

Policy information about studies involving animals; ARRIVE guidelines recommended for reporting animal research

Laboratory animals	C57BL/6 mice

Wild animals	N.A.
Field-collected samples	N.A.
Ethics oversight	Housing and experimentation was carried out in accordance with the Animal (Scientific Procedures) Act 1986 and current guidelines approved by the Ethical Review Committee of Queen's University (Belfast, UK).

Note that full information on the approval of the study protocol must also be provided in the manuscript.
